# Secreted Sulfatases *Sulf1* and *Sulf2* Have Overlapping yet Essential Roles in Mouse Neonatal Survival

**DOI:** 10.1371/journal.pone.0000575

**Published:** 2007-06-27

**Authors:** Charles R. Holst, Hani Bou-Reslan, Bryan B. Gore, Karen Wong, Deanna Grant, Sreedevi Chalasani, Richard A. Carano, Gretchen D. Frantz, Marc Tessier-Lavigne, Brad Bolon, Dorothy M. French, Avi Ashkenazi

**Affiliations:** 1 Department of Molecular Oncology, Genentech, Inc., South San Francisco, California, United States of America; 2 Department of Biomedical Imaging, Genentech, Inc., South San Francisco, California, United States of America; 3 Department of Research Drug Discovery, Genentech, Inc., South San Francisco, California, United States of America; 4 Department of Molecular Biology, Genentech, Inc., South San Francisco, California, United States of America; 5 Department of Pathology, Genentech, Inc., South San Francisco, California, United States of America; 6 Graduate Program in Neurosciences, Stanford University, Stanford, California, United States of America; 7 GEMpath, Inc., Cedar City, Utah, United States of America; Baylor College of Medicine, United States of America

## Abstract

**Background:**

Heparan sulfate proteoglycans (HSPGs) use highly sulfated polysaccharide side-chains to interact with several key growth factors and morphogens, thereby regulating their accessibility and biological activity. Various sulfotransferases and sulfatases with differing specificities control the pattern of HSPG sulfation, which is functionally critical. Among these enzymes in the mouse are two secreted 6-*O*-endosulfatases, *Sulf1* and *Sulf2*, which modify HSPGs in the extracellular matrix and on the cell surface. The roles of *Sulf1* and *Sulf2* during normal development are not well understood.

**Methods/Results:**

To investigate the importance of *Sulf1* and *Sulf2* for embryonic development, we generated mice genetically deficient in these genes and assessed the phenotypes of the resulting secreted sulfatase-deficient mice. Surprisingly, despite the established crucial role of HSPG interactions during development, neither *Sulf1*- nor *Sulf2*-deficient mice showed significant developmental flaws. In contrast, mice deficient in both *Sulf1*and *Sulf2* exhibited highly penetrant neonatal lethality. Loss of viability was associated with multiple, although subtle, developmental defects, including skeletal and renal abnormalities.

**Conclusions:**

These results show that *Sulf1* and *Sulf2* play overlapping yet critical roles in mouse development and are redundant and essential for neonatal survival.

## Introduction

Normal developmental processes of metazoans depend upon cell-cell communication, which is mediated by a diverse yet highly conserved set of secreted protein factors. Genetic analysis of embryonic development in *C. elegans, Drosophila*, zebrafish, and mouse has revealed the biological importance of heparan sulfate proteoglycans (HSPGs) in regulating the activity of multiple secreted growth factors and morphogens in various developmental processes [1], [2]. Among these factors are the ligands of the fibroblast growth factor (FGF), bone morphogenetic protein (BMP), wingless/WNT, and Hedgehog (Hh) pathways. HSPGs have been shown to regulate cell signaling positively or negatively, depending on pathway and context.

HSPGs such as glypicans, syndecans, agrins, and perlecans are proteins modified with heparan sulfate during their biosynthetic transit through the Golgi apparatus. Following the addition of a tetrasaccharide linker to specified serines in the polypeptide backbone, the exostosin (EXT) enzymes catalyze the addition of unbranched repeats of the glucuronic acid, *N*-acetylglucosamine disaccharide [3], [4]. The uniformity of this simple disaccharide repeat is then diversified by the activity of several other biosynthetic enzymes, including sulfotransferases with differing specificities, *N*-deacetylase/*N*-sulfotransferases (NDSTs), and an epimerase, eventually producing a polysaccharide sidechain of astonishing complexity [5], [6]. The sulfotransferases catalyze the addition of sulfate to the *N*, 3-*O*, and 6-*O* positions of *N*-acetylglucosamine and the 2-*O* position of iduronic acid. The degree and specific pattern of sulfation is determined, in a tissue-specific manner, by the expression of these biosynthetic enzymes [7]. The sulfation pattern of HSPGs is critical for determining the specificity and affinity of binding to growth factors and morphogens [5].

Heparan sulfate modification of proteins is essential for mouse development. Embryos deficient for the *Ext1* glycosyltransferase, and hence devoid of nearly all heparan sulfate, die during gastrulation [8]. The relevance of sulfation pattern of HSPGs to normal development has been underscored by the analysis of the neonatal lethality and pleiomorphic phenotypes of *Ndst1*- and *Hs2st*-deficient mice [9]–[11]. Whereas *Ndst1*-deficient embryos exhibit cerebral and pulmonary hypoplasia and craniofacial defects, *Hs2st*-deficient embryos show cerebral and renal hypoplasia accompanied by skeletal and eye defects. Furthermore, inhibition of the different 6-*O*-sulfotransferase genes has profound effects on muscle and vascular development in zebrafish [12], [13].

An additional regulatory level of HSPG sulfation status was recently suggested by the discovery and cloning of a novel class of secreted sulfatase enzymes [14], [15]. These proteins, named *Sulf1* and *Sulf2* in the mouse, can act on HSPGs on the cell surface as well as within the Golgi apparatus, thereby affecting sulfation status *ex post facto* [15], [16]. The hydrolytic activity of these enzymes is specific to the 6-*O*-sulfate of *N*-acetylglucosamine [15], [17]. Because these sulfatases are found in soluble as well as cell surface-associated forms [14], [15], they also may possess non-cell autonomous or paracrine activity.

Given the critical role of regional HSPG sulfation patterns during development, we sought to assess the importance of *Sulf1* and *Sulf2* during mouse development. To this end, we generated mouse strains with disruption of the *Sulf1* or *Sulf2* genes. We report here that deficiency in either *Sulf1* or *Sulf2* alone did not appreciably affect normal developmental or physiological processes. In contrast, disruption of both *Sulf1* and *Sulf2* simultaneously resulted in high-penetrance perinatal lethality in conjunction with multiple subtle developmental defects. These results indicate that *Sulf1* and *Sulf2* play overlapping and essential role(s) during development. Our results confirm and expand upon the recently reported phenotypes of sulfatase-deficient mice by Lamanna *et al*. [17], [18], but differ from the phenotypes of the *Sulf2*-deficient animals described by Lum *et al*. [19].

## Materials and Methods

The Institutional Animal Care and Use Committee approved all protocols used in this study.

### *Sulf1* and *Sulf2* targeting

Embryonic stem (ES) cells with retroviral gene trap vectors inserted into the *Sulf1* (OST352220) and *Sulf2* (OST311938) loci were generated in collaboration with Lexicon Genetics (The Woodlands, TX) using methods described previously [20]. *Sulf1* was disrupted by insertion of the VICTR48 construct; this allele is therefore named *Sulf1^Gt(VICTR48)352220Lex^*. *Sulf2* was disrupted by the VICTR37 retroviral construct, which included a promoterless β-galactosidase cassette in addition to the Neomycin resistance gene. The *Sulf2* allele generated by the insertion mutation is named *Sulf2^Gt(VICTR37)311938Lex^*. The insertion sites were determined by sequence analysis. The *Sulf1*- or *Sulf2*-targeted 129/SvEvBrd ES cells were microinjected into blastocysts and implanted into C57BL/6J females. Germline transmission was assessed in F1 pups by PCR analysis of genomic DNA. Heterozygous mice were either intercrossed to assess the phenotypes on a mixed strain background or backcrossed onto the C57BL/6N background. Unless otherwise specified, the results presented herein were performed using the N4 (*Sulf2*), or N5 or N6 (*Sulf1*) backcross generations. For simplicity's sake, we refer to the gene trap alleles of the genes as *Sulf1* ^−^ and *Sulf2* ^−^ and the wild-type alleles as *Sulf1* ^+^ and *Sulf2* ^+^.

### Generation of mice deficient for both *Sulf1* and *Sulf2*

*Sulf1* ^−/−^ (N3, C57BL/6N) mice were crossed to *Sulf2* ^−/−^ (N2, C57BL/6N) mice to generate F1 *Sulf1* ^+/−^ *Sulf2* ^+/−^ mice. F1 mice were intercrossed to assess the viability of mice with the different possible genotypes. To generate sufficient *Sulf1* ^−/−^ *Sulf2* ^−/−^ mice to study, we routinely intercrossed F2 *Sulf1* ^+/−^ *Sulf2* ^−/−^ to *Sulf1* ^−/−^ *Sulf2* ^+/−^ mice to produce study cohorts with *Sulf1* ^+/−^ *Sulf2* ^+/−^ mice as controls.

### Genotyping of *Sulf1* and *Sulf2* alleles

We utilized a PCR genotyping strategy to detect the wild type and disrupted alleles of *Sulf1* and *Sulf2*. Tail DNA was prepared using the Extract-N-Amp extraction kit following the manufacturer's protocol (Sigma, St. Louis, MO). PCR conditions were as follows: 94°C for 4 min., 1 cycle; 94°C for 60 s, 60°C for 30 s, 72°C for 60 s (30–35 cycles); 72°C for 10 min., 1 cycle. For genotyping of tails from neonate and young adult mice, 30 cycles were sufficient. We increased the cycle number to 35 when detecting DNA from embryonic tissue.

The retroviral insertion in the *Sulf1* locus was detected using primers Sulf1-R1 and LTR, which generated a 334-bp product. We detected the wild type *Sulf1* allele using primers Sulf1-F and Sulf1-R1, which produced a 459-bp amplicon. Using a similar strategy, the insertion allele in the *Sulf2* locus were detected using primers Sulf2-R and LTR2, producing a 440-bp band; the wild-type *Sulf2* allele was amplified with primers Sulf2-F2 and Sulf2-R, making a 759-bp product. The appropriate three-primer sets were used simultaneously without evidence of interference when evaluating both modified genes. *Sulf1*; *Sulf2* mice were genotyped as above, amplifying the *Sulf1* and *Sulf2* loci in separate reactions.

Sequences of genotyping primers were as follows, from 5′ to 3′: LTR, ATA AAC CCT CTT GCA GTT GCA TC; Sulf1-F, CCG CAA AGA CTT GGA ATT AAC TC; Sulf1-R1, CTT CAC ACA CTC CAC ACT CAG TTC T; LTR2, AAA TGG CGT TAC TTA AGC TAG CTT GC; Sulf2-F2, TTG ACT TTC TGG GGA GGG TGG ATG; and Sulf2-R, GAT GGG CCA CTC CTG AGA TAA CCT G.

### RNA purification and quantitative RT-PCR

For quantitative real-time RT-PCR (Q-RT-PCR), total RNA was isolated using the RNeasy kit essentially according to the manufacturer's protocol, including the on-column DNase digestion (Qiagen, Valencia, CA). Immediately prior to column purification, frozen tissue was homogenized in RLT buffer containing β-mercaptoethanol using a stator homogenizer followed by a QiaShredder column (Qiagen). To remove all contaminating genomic DNA, we re-purified the eluted RNA over a fresh RNeasy column, including a second DNase treatment. This routinely reduced the amplification of a control reaction omitting reverse transcriptase to below the limit of detection, allowing us to conclude that signal was indeed due to RNA and not contaminating genomic DNA.

Q-RT-PCR was done according to the *Taqman* protocol (Applied Biosystems, Foster City, CA). Using 100 ng total RNA per 50 µl reaction, we detected and quantitated the amplification products using an ABI 7500 system (Applied Biosystems), normalizing to either the GAPDH or RPL19 housekeeping genes. Relative changes in gene expression were quantitated using the 2^−ΔΔCt^ method [21]. Primer/probe sets used were as follows, with forward primers denoted by F, reverse primers by R, and FAM/TAMRA-labeled probes as T: Sulf1-4535F, CCT CGA CGT GCT AAA CTT GA; Sulf1-4611R, TAT TCC CGC AGG ATT TAT TTC; Sulf1-4556T, TAG CAG AAA GGC ATG GCT CAC AAT G; Sulf2-3364F, CCC TTG AGC TTT CAG ACA TTT; Sulf2-3428R, CAG TTC TGG GAT GGA TAA CAA A; Sulf2-3386T, TTC CTG CCC GGG ATT CGT TC; RPL19-F2, AGA AGG TGA CCT GGA TGA GAA; RPL19-R2, TGA TAC ATA TGG CGG TCA ATC T; RPL19-T2, CTT CTC AGG AGA TAC CGG GAA TCC AAG; GAPDH-F, ATG TTC CAG TAT GAC TCC ACT CAC G; GAPDH-R, GAA GAC ACC AGT AGA CTC CAC GAC A; and GAPDH-T, AAG CCC ATC ACC ATC TTC CAG GAG CGA GA.

### Whole-mount embryo RNA *in situ* hybridization

Embryos were harvested at E8.5-E12.5 (with noon of the day the vaginal plug was observed designated as E0.5) and fixed in freshly prepared 4% paraformaldehyde (PFA) in PBS (pH 7.4) overnight at 4°C. Primers used to amplify the *Sulf1* transcript from a mouse embryo cDNA library to TOPO-clone into pCR-II-TOPO (Invitrogen, Carlsbad, CA) were Sulf1-wmISH-S, ACC AGT CAG CCA GAG CGT and Sulf1-wmISH-AS, CTT CCC ATC CAT CCC ATA ACT. Following sequencing to ensure fidelity of the amplification and orientation of insertion, we generated antisense, DIG-labeled probe from linearized plasmid template using the mMESSAGE mMACHINE kit (Ambion, Austin, TX), following the manufacturer's instructions. Hybridization and detection of DIG-labeled probe were done according to standard protocols.

### Section RNA *in situ* hybridization

Following fixation in freshly prepared 4% PFA in PBS (pH 7.4) overnight at 4°C, we equilibrated embryos (E9.5–E12.5) overnight in 30% sucrose in PBS, followed by 1 h in Tissue-Tek O.C.T. (Sakura). Specimens then were embedded in fresh O.C.T. and frozen in an ethanol/dry ice bath. *In situ* hybridization was carried out on 20-µm transverse sections of wild type embryos. To generate ^35^S-radiolabeled RNA probe for *in situ* hybridization, we used the mRNA*locator* kit (Ambion) according to the included instructions. The template for the probe synthesis was a PCR product with a T7 promoter included in the primer, using the following primers: Sulf1-sISH-S, TAT CCG GTG CAA GCA ACA T; Sulf1-sISH-T7-AS, TAA TAC GAC TCA CTA TAG GGA GGC TGT TCA GAT GCA GGG TTT G; Sulf2-sISH-S, CCC ACA ACT TCC TCT TCT GC; and Sulf2-sISH-T7-AS, TAA TAC GAC TCA CTA TAG GGA GGC CCA TAG CTG TCC CAG TGA T.

*In situ* hybridization using DIG-labeled probes specific to *Collagen Type X* and *Collagen Type II* was performed on paraformaldehyde-fixed, paraffin-embedded tissue, as previously described [22].

### β-Galactosidase expression pattern analysis

All solutions were made in 0.1 M phosphate buffer (pH 7.3). Embryos (E10.5–E12.5) were washed in unadulterated buffer and then fixed for 30–45 min in buffer containing 0.2% glutaraldehyde, 5 mM EGTA, and 2 mM MgCl_2_. Embryos were then washed 3×15 min in buffer containing 2 mM MgCl_2_, 0.01% deoxycholate, 0.02% Nonidet-P40 and stained in the dark for 1–12 hours at room temperature in buffer containing 5 mM K_3_Fe(CN)_6_, 5 mM K_4_Fe(CN)_6_, 2 mM MgCl_2_, 0.02% NP-40, and 1 mg/ml 5-bromo-4-chloro-3-indolyl-β-D-galactopyranoside (X-gal, IBI Shelton Scientific, Peosta, IA). Embryos were then washed in PBS (pH 7.4) and post-fixed in neutral buffered 10% formalin (NBF). Stained specimens were evaluated as whole mounts and then, in selected instances, in 12-µm-thick transverse cryosections of O.C.T.-embedded material.

### Skeletal preparations

Near-term (E18.5) fetuses or neonates were killed, flayed, eviscerated, and fixed in 100% ethanol for 1–2 days, followed by an overnight acetone treatment. Specimens were stained in 0.015% alcian blue, 0.005% alizarin red in 5% acetic acid, 60% ethanol for 2–3 days at room temperature; washed briefly in tap water; then cleared in 1% KOH for several days. Cleared specimens were passed through a series of glycerol solutions of increasing concentration prior to visualization on a Zeiss dissecting microscope.

### Micro-computed tomography (µCT) image acquisition

The mouse embryos were imaged with a µCT40 (SCANCO Medical, Basserdorf, Switzerland) x-ray micro-computed tomography system. A sagittal scout image, comparable with a conventional planar x-ray, was obtained to define the start and end point for the axial acquisition of a series of µCT image slices. The location and number of axial images were chosen to provide complete coverage of the embryo. The embryos were imaged with air as the background media. The µCT images were generated by operating the x-ray tube at an energy level of 45 kV, a current of 177 µA and an integration time of 300 milliseconds. Axial images were obtained at an isotropic resolution of 16 µm.

### Analysis of µCT images

The relationship between the image intensity values and bone mineral density was assumed to be linear and was obtained by scanning a 97% pure hydroxyapatite (HA) sample (2.91 gHA/cm^3^). Three-dimensional (3D) surface renderings were created from the µCT data with the use of Analyze (AnalyzeDirect Inc., Lenexa, KS), an image analysis software package. The bone surface renderings were generated by applying a bone mineral density of threshold of 0.54 gHA/cm^3^. The bones of interest (femur, humerus, supraoccipital, interparietal, parietal, frontal, basisphenoid and basioccipital) were extracted for quantitative analysis by the applying a series of image analysis steps (thresholding, morphological filtering and region growing operations) to the volumetric image data. For the long bones, the distance between the ends of the bone was measured as a straight line and an average was computed using the contralateral bone measurements. The volume (mm^3^) and mean bone density (gHA/cm^3^) of the defined bones was measured and an average was obtained for the ipsilateral and contralateral bones for each embryo.

### Necropsy and histological preparation of conceptuses and neonates

Fetuses (E15.5 and E18.5) were removed from the uteri of anesthetized dams. The tip of each fetus's tail was harvested for genotyping by PCR, after which the fetus and placenta were fixed by immersion in either 4% PFA, 10% NBF, or Bouin's solution. Some fetuses were bisected longitudinally just to the left of the midline, while others were decapitated so that the heads could be cut coronally. A few neonates (including placentas) were handled in a similar manner. Fixed tissues were processed into paraffin using a conventional protocol. Serial 5-µm-thick coronal sections of each head block were acquired at several steps, each separated by a distance of 250 µm. Serial 5-µm-thick sections of each torso block were acquired at several steps, each separated by a distance of 500 µm. Serial sections at each step were stained with hematoxylin and eosin (H&E) or cresyl violet (to better delineate subcellular characteristics of neural cells). Lesions were scored (with foreknowledge of the fetus's genotype) using a tiered, semi-quantitative grading scale: absent, minimal, mild, moderate, marked. Subsequently, a coded (“blinded”) histopathological assessment for a given organ was performed by inverting all slides and then sorting them into groups using criteria established in the uncoded examination.

### Mouse embryonic fibroblast isolation

E12.5 embryos were dissected from the extraembryonic tissues in PBS, then the liver and head were removed. After mincing with scissors, the remaining tissue was digested in 0.1% trypsin/EDTA in PBS for 20 min at 37°C, with mixing every 5 min. After washing twice with culture medium (DMEM with 10% fetal bovine serum (FBS), 2 mM L-glutamine, and penicillin/streptomycin), the cells from a single embryo were plated in a 25-cm^2^ flask. One day later, cells were trypsinized, counted, and either frozen in aliquots or used immediately for signaling experiments.

### FGF signaling in embryonic fibroblasts

First- or second-passage fibroblasts were serum-starved overnight in assay medium containing 0.5% FBS at a density of 10^5^ cells per well of a 6-well plate. The following day, prewarmed FGF1- or FGF2-containing assay medium (or assay medium lacking FGF) was added to the cells, which were then returned to the 37°C incubator for 10 minutes. Cells were lysed for 15 minutes at 4°C, with rocking, in 1×RIPA buffer (Upstate) supplemented with Complete protease inhibitors (Roche), 2 mM sodium orthovanadate, and 5 mM sodium fluoride. Lysates were cleared by centrifugation (10 minutes at 11,000 *g*), then prepared for electrophoresis using LDS buffer and DTT (Invitrogen). Equal amounts of protein were loaded per lane of a 4–12% Bis-Tris NuPAGE gel and then separated according to the manufacturer's recommended protocol (Invitrogen). After transferring the protein to nitrocellulose membranes, non-specific binding was blocked using 5% milk in Tris-buffered saline solution containing 0.2% Tween-20 (TBST). Primary antibodies were incubated overnight at 4°C in 1% BSA in TBST (Pierce). Peroxidase-conjugated secondary antibodies were used for 1 hour at room temperature. Peroxidase activity was detected using ECL Plus (GE Healthcare). Antibodies and dilutions used: Phospho-FRS2, 1∶1000 (Upstate); Total FRS2, 1∶1000 (Upstate); anti-rabbit-HRP, 1∶10,000 (Amersham). Bands were quantitated using the integrated density function of ImageJ. Data shown are representative of three independent experiments.

### Statistics

Histopathologic (ordinal) data from the blinded microscopic evaluation were assessed using the non-parametric chi-square test. Nominal data were analyzed by two-tailed Student's *t*-test. All calculations were done using commercial JMP statistical software (v. 5.0; SAS Institute, Cary, NC), and significance assigned to p-values ≤0.05.

## Results

### Targeted disruption of *Sulf1*

**Figure 1:**
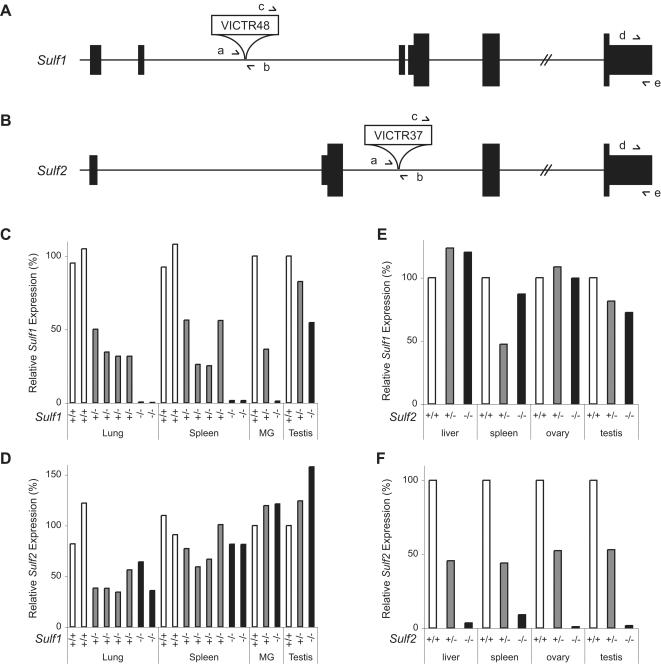
*Sulf1* and *Sulf2* gene trap alleles are hypomorphic. (A) *Sulf1* retroviral insertion gene trap allele. The VICTR48 retrovirus inserted into position 12,733,618 of chromosome 1 (February 2006 (mm8) assembly of the mouse genome, UCSC Genome Browser), which corresponds to intron 2 of *Sulf1*. Primers *a*, *b*, and *c* correspond to Sulf1-F, Sulf1-R1, and LTR, respectively, and were used to genotype the mice. Primers *d* and *e* in the 3′ untranslated region refer to Sulf1-4535F and Sulf1-4611R, which were used for Q-RT-PCR amplification of the *Sulf1* transcript. The position of the open reading frame is indicated by the taller boxes; the untranslated region is represented by the shorter boxes. Not to scale. (B) *Sulf2* retroviral gene trap allele. The VICTR37 retrovirus inserted into position 165,818,084 of chromosome 2, which is intron 1 of *Sulf2*. Primers *a, b*, and *c* correspond to Sulf2-F2, Sulf2-R, and LTR2, respectively, and were used to genotype the mice. Primers *d* and *e* in the 3′ untranslated region refer to Sulf2-3364F and Sulf2-3428R, which were used for Q-RT-PCR amplification of the *Sulf2* transcript. (C and D) Q-RT-PCR analysis of *Sulf1* (C) and *Sulf2* (D) in tissues from mice with different *Sulf1* genotypes (white bars, *Sulf1* ^+/+^; grey bars, *Sulf1* ^+/-^; black bars, *Sulf1* ^-/-^). The Cycle to Threshold (Ct) values for duplicate or triplicate samples were averaged. Each bar represents a single animal. After normalization to GAPDH or RPL19, relative *Sulf1* expression levels (compared to an average of the two WT littermate controls analyzed) were calculated based upon the 2^-ΔΔCt^ method [21]. MG refers to mammary gland. (E and F) Q-RT-PCR analysis of *Sulf1* (E) and *Sulf2* (F) expression in tissues from *Sulf2* ^+/+^, *Sulf2* ^+/-^, or *Sulf2* ^-/-^ adult mice. Analysis was done as described above for the *Sulf1* mice. Abbreviation used: MG refers to mammary gland tissue.

An ES cell clone with a retroviral insertion in the intron between exons 2 and 3 of the *Sulf1* gene (using the nomenclature and gene structure information provided in Morimoto-Tomito *et al*.)[15] was identified by screening a gene trap ES cell library (Fig. 1A). Chimeric mice carrying this targeted *Sulf1* allele were generated using standard techniques. Upon intercrossing the first F1 generation of *Sulf1* ^+/−^ mice in the mixed genetic background, we observed no deviation from expected Mendelian birth ratios (Table 1). Both male and female homozygous mutant (*Sulf1* ^−/−^) mice were capable of generating offspring, indicating no obvious fertility defect (data not shown). Similar viability and fertility results were obtained with *Sulf1* ^−/−^ mice after backcrossing the mutant allele 5 times onto the C57BL/6N strain (N5). *Sulf1* ^−/−^ mice were therefore viable and fertile in both a mixed 129/SvEvBrd; C57BL/6 background and a purer C57BL/6 genetic background.

**Table 1:** ^−/−^ and *Sulf2* ^−/−^ mice are present at Mendelian frequencies.

Parental Genotype	Offspring Genotype						χ^2^	*df*	*p*
	Observed (%)			Expected					
	+/+	+/−	−/−	+/+	+/−	−/−			
*Sulf1* (N5) +/−×+/−	86 (27)	168 (53)	65 (20)	79.75	159.5	79.75	3.67	2	NS
*Sulf2* (N0) +/−×+/−	11 (23)	21 (44)	16 (33)	12	24	12	2.25	2	NS
*Sulf2* (N4) +/−×+/−	43 (30)	65 (46)	33 (23)	35.25	70.5	35.25	1.79	2	NS

NS means not significant (*p*>0.05).

As the retroviral insertion site is located in an intron upstream of the exon containing the ATG start codon of *Sulf1* (Fig. 1A), we suspected the allele might be hypomorphic rather than null. To confirm whether the retroviral insertion indeed resulted in a loss- or reduction-of-function allele, we performed quantitative RT-PCR (Q-RT-PCR) using primers designed to amplify the 3′ UTR of *Sulf1*. We found that the retroviral insertion resulted in a severe hypomorphic allele in nearly all tissues examined. Whereas heterozygous animals showed the expected ∼50% decrease in *Sulf1* expression, homozygotes had between 0.2% and 1.7% of *Sulf1* mRNA levels relative to wild-type control tissues (Fig. 1C). Interestingly, we observed no substantial decrease in expression of the *Sulf1* message in RNA derived from the testis (Fig. 1C). It has been shown previously that testis tissue produces a truncated *Sulf1* transcript [23], consistent with the use of an alternative promoter that might be unaffected by the presence of the genetrap retroviral insertion. Finally, we did not observe any significant change in *Sulf2* expression in the tissues derived from the *Sulf1* ^−/−^ mice (Fig. 1D).

The hypomorphic nature of the *Sulf1* ^−^ allele was further verified by immunohistochemistry. Sections of wild-type (WT) and *Sulf1* ^−/−^ embryonic tissue were stained with a Sulf1-specific rabbit polyclonal antibody. Compared to WT embryos, *Sulf1* ^−/−^ embryos exhibited a profound loss of expression of the Sulf1 protein in the floor plate of the neural tube, a particularly prominent site of Sulf1 expression (Fig. S1). Along with the quantitative RT-PCR results (Fig. 1C), these data confirm that the *Sulf1* genetrap allele is a severe hypomorph, at the mRNA and protein level.

### Embryonic and adult *Sulf1* expression

We assessed the expression of *Sulf1* during development by *in situ* hybridization (ISH) in whole mounts and tissue sections of embryos from stages E9.5 to E12.5 (Fig. S2 and data not shown). Reminiscent of the expression pattern of the rat *Sulf1* homolog (also known as RSulfFP1)[24], we detected mouse *Sulf1* expression in the floor plate of the midbrain, hindbrain, and neural tube, the choroid plexus, the clefts of the branchial arches, dorsal telencephalon, somites, eyes, palate, tongue, nasal pits, the forelimb arm girdle, and the condensing mesenchyme in the distal limb buds (Fig. S2). ISH on sagittal sections of E15 embryos and E17 fetuses revealed continued expression in the choroid plexus and the cartilaginous anlagen of the digits, as well as scattered expression in the lungs, gastrointestinal tract, brown adipose tissue, salivary gland, and the esophageal lining (data not shown).

Section-based ISH of a panel of adult tissues revealed expression in the photoreceptor layer of the retina, the Harderian gland, blood vessels and some tubules in kidney, blood vessels in the lungs, submucosa of the stomach, and intestinal mucosal epithelium (data not shown). Microarray analysis of an extensive panel of normal adult mouse tissues revealed detectable expression of *Sulf1* in nearly all sites examined, with uterus, bladder, mesenteric lymph nodes, prostate, esophagus, lung, mammary gland, and bone showing particularly strong expression (Fig. S3).

### Targeted disruption of *Sulf2*

From a random insertion ES cell library, a clone was isolated with an insertion between exon 2 (which contains the ATG start codon) and exon 3 of the *Sulf2* gene (Fig. 1B). Heterozygous chimeric mice were isolated and bred to generate *Sulf2* mutant animals. In contrast to a recently published report [19], we observed no deviation from the expected Mendelian birth ratios of homozygous mutant animals, neither in the original mixed 129/SvEvBrd; C57BL/6 background nor in mice at the N4 generation of backcrossing into the C57BL/6N strain (Table 1). We also observed that *Sulf2* ^−/−^ males and females were fertile (data not shown), indicating that *Sulf2* is not necessary for reproduction.

We verified that the retroviral insertion into the *Sulf2* locus also resulted in a hypomorphic allele by Q-RT-PCR. Similar to our results in the *Sulf1*-disrupted mice, we found ∼50% reduction in *Sulf2* mRNA levels in tissues derived from *Sulf2* heterozygotes but a marked decrease (0.9–9.1% of WT levels, depending on the tissue examined) in *Sulf2* expression in tissue from *Sulf2* ^−/−^ homozygotes (Fig. 1F). We did not observe a compensatory change of *Sulf1* expression in *Sulf2* mutants (Fig. 1E).

### Embryonic and adult *Sulf2* expression

The retroviral gene trap cassette used to disrupt *Sulf2* contained a β-galactosidase reporter, which we used to assess the *Sulf2* expression pattern during development. When detecting β-galactosidase activity in a litter of embryos from a *Sulf2* ^+/−^ intercross, the intensity of staining corresponded to the genotype status as determined by PCR of genomic DNA (Fig. S4). *Sulf2* is expressed in a dynamic pattern at all developmental stages tested (E10.5–E12.5; Figs. S4 and S5). Sectioning the stained embryos, we detected β-galactosidase activity in the floor plate of the hindbrain, floorplate and ventral progenitor region of the neural tube, choroid plexus, roof plate of the forebrain and midbrain, the developing retina, mesenchyme of the head and branchial arches, dorsal root ganglia, somites (dermomyotome), forelimb buds (in a posterior-to-anterior gradient), hindlimbs (more uniform intensity), the caudal notochord, and the right ventricle of the heart (data not shown).

ISH of transverse sections through the forelimb level of E11.5–E12.5 embryos confirmed the veracity of the β-galactosidase staining, but also revealed additional expression in the condensing sclerotome ventral to the neural tube; mesenchyme surrounding the dorsal aorta, sinus venosus, and left common cardinal vein; the lining of the esophagus; mesenchymal cells surrounding the bronchi in the lung buds; palate and tongue; and condensing mesenchyme surrounding the presumptive digits in the distal limbuds (Fig. S4).

We also detected β-galactosidase activity by gross observation in a panel of normal tissues from an adult *Sulf2* ^−/−^ animal. Activity was undetectable in muscle and spleen. Limited expression was detected in the lung (limited to the cells lining the large bronchial airways), stomach (cells on serosa of stomach, particularly in the pyloric region), and uterus (subset of cells in both the myometrium and endometrium). Abundant activity was also revealed in the ovary and testis (data not shown). Consultation of a recently published microarray analysis of an extensive panel of normal mouse tissues [25] revealed detectable expression of *Sulf2* in a wide variety of tissues examined, with ovary, umbilical cord, uterus, adipose, bladder, and intestine showing particularly strong expression (Fig. S5). *Sulf2* was also expressed in a variety of brain structures, including the hippocampus, cerebral cortex, and hypothalamus (Fig. S5).

### Absence of major abnormality in *Sulf1* ^−/−^ or *Sulf2* ^−/−^ mice

Neither *Sulf1* ^−/−^ nor *Sulf2* ^−/−^ mice had any defect in long-term survival, with both males and females living to >1.5 years of age. As described above and previously, *Sulf1* and *Sulf2* are expressed in a variety of embryonic and adult tissues (Fig. 1; Figs. S1, S2, S3, S4, S5)[19], [24], [26]. We therefore cast a wide net in searching for phenotype(s) in the *Sulf1* ^−/−^ and *Sulf2* ^−/−^ mice. We observed no gross differences in appearance or behavior between wild-type, heterozygous and single mutant mice. Aged adult mice were subjected to necropsy, at which time we performed gross and histological analysis on a subset of organs, including lung, liver, stomach, small intestine, colon, brain, eye, spleen, kidney, and heart. Apart from common age-related changes, which were observed in both control and sulfatase-deficient animals, no notable gross or histological changes were found in the single mutants (data not shown). We conclude that neither the *Sulf1*- nor the *Sulf2*-deficient animals exhibited any obvious gross or histological abnormalities.

**Figure 2:**
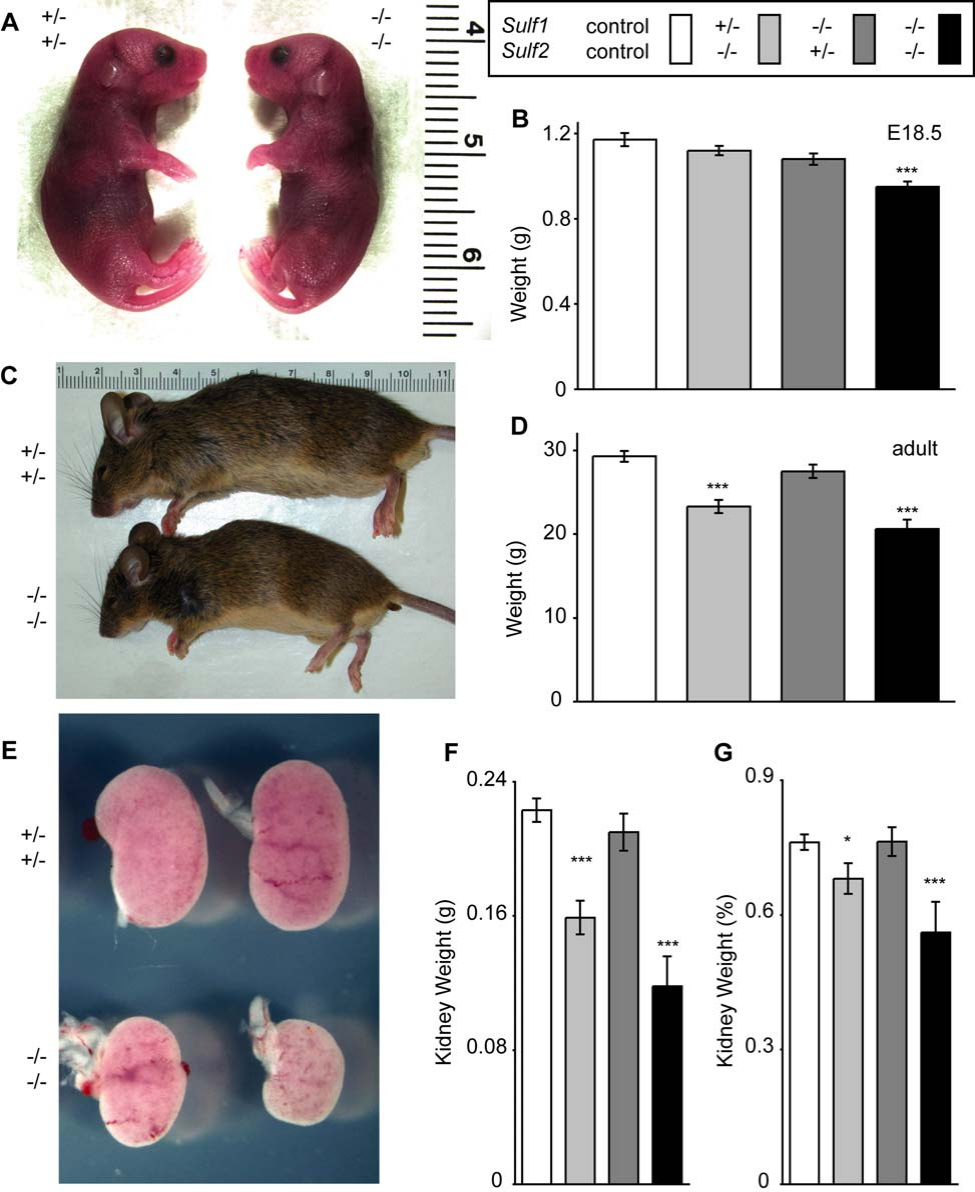
*Sulf1* ^-/-^ *Sulf2* ^-/-^ mice have decreased body and kidney weights at birth and (for the few that survive) as adults. (A) Representative image of *Sulf1* ^-/-^ *Sulf2* ^-/-^ and littermate control E18.5 day embryos just after birth, but prior to feeding. Note the pink skin-tone and apparently normal vascularization and circulation. (B) Quantification of embryo weight (mean±SEM) at E18.5. *Sulf1* ^-/-^ *Sulf2* ^-/-^ embryos show a 19% decrease in weight. These embryos also have a 12% decreased crown-rump length (data not shown). Number of embryos analyzed were as follows: Control (pooled ^+/+^;^+/+^, ^+/-^;^+/+^, ^+/+^;^+/-^, and ^+/-^;^+/-^ embryos), *n* = 31; *Sulf1* ^+/-^ *Sulf2* ^-/-^, *n* = 32; *Sulf1* ^-/-^ *Sulf2* ^+/-^, *n* = 26, and *Sulf1* ^-/-^ *Sulf2* ^-/-^, *n* = 24. (C) Representative image of a typical small *Sulf1* ^-/-^ *Sulf2* ^-/-^ 10-week-old male and a control littermate. Note the proportionate small stature and the lack of gross morphological differences. (D) Body weights (mean±SEM) of 15-week-old males. Weights of *Sulf2* ^-/-^ and *Sulf1* ^-/-^ *Sulf2* ^-/-^ mice were ∼20% and ∼30% smaller, respectively, than controls. Number of adult males analyzed were as follows: Controls (*Sulf1* ^+/-^ *Sulf2* ^+/-^), *n* = 26; *Sulf1* ^+/-^ *Sulf2* ^-/-^, *n* = 8; *Sulf1* ^-/-^ *Sulf2* ^+/-^, *n* = 10, and *Sulf1* ^-/-^ *Sulf2* ^-/-^, *n* = 5. (E, F, G) Kidneys of *Sulf1* ^-/-^ *Sulf2* ^-/-^ mice were small at E18.5 (E) and remain disproportionately small (mean±SEM) into adulthood (F, G). The *Sulf2* ^-/-^ mutants also had kidneys of decreased size, though the degree of reduction was more proportional to these animals' decrease in body weight. The weights of 15-week-old adult male kidneys are represented as a percentage of total body weight in panel G. The kidneys analyzed here are from the same cohort of animals whose body weights were presented in panel D. Statistical significance was evaluated using a two-tailed Student's *t*-test; *, *p*<0.05. ***, *p*<0.001.

Whereas *Sulf1* ^−/−^ animals had normal body weights from birth to adulthood (Fig. 2), we did observe a slight decrease in body weight in 16–18 month-old *Sulf2* ^−/−^ females (in the 129SvEvBrd; C57BL/6 mixed background), with control animals weighing 39.6±3.7 g (*n* = 4, mean±SEM) and *Sulf2* ^−/−^ females weighing 24.6±1.6 (38% decrease; *n* = 3, *p* = 0.02 by two-tailed Student's *t*-test). In this same cohort of aged mice, however, the *Sulf2* ^−/−^ males (34.2±2.7 g, *n* = 5) had indistinguishable weights from their littermate controls (36.1±0.5 g, *n* = 3; *p*≈0.6 by *t*-test). A larger cohort of younger *Sulf2* ^−/−^ male mice, age ∼14–15 weeks, showed a statistically significant reduction in body weight (Fig. 2D), with control animals weighing 29.3±0.6 *g* (*n* = 26) and *Sulf2* ^−/−^ animals weighing 23.3±0.8 *g* (20% decrease, *n* = 8; *p*<0.0001 by *t*-test). Notably, when *Sulf2* ^−/−^ adult mice did exhibit decreased body weight, all organs examined except the kidneys showed a proportional decrease in weight (Fig. 2 and data not shown). In summary, although *Sulf2* ^−/−^ adults had decreased body weight, they appeared grossly, histologically, and behaviorally normal.

### *Sulf1* ^−/−^ *Sulf2* ^−/−^ double-knockout mice show perinatal lethality

Given that the single mutant mice exhibited very mild or no phenotype, we postulated that the two secreted sulfatases perform redundant function(s) *in vivo*. To examine this hypothesis, we crossed the *Sulf1* and *Sulf2* mutant mice to generate compound heterozygotes. *Sulf1* ^+/−^ *Sulf2* ^+/−^ siblings were then intercrossed, with the expectation that 1/16^th^ of their offspring would have the *Sulf1* ^−/−^ *Sulf2* ^−/−^ genotype. Interestingly, genotyping at postnatal day 9.4±3.7 (mean±SD), we observed only ∼18% of the expected number of double mutants (11 expected, 2 observed; Table 2). This reduction in viability demonstrates that the two secreted sulfatases perform redundant and essential function(s) *in vivo*.

**Table 2:** *Sulf1* ^−/−^ *Sulf2* ^−/−^ mice exhibit neonatal lethality.

Parental Genotypes^1^		Offspring Genotypes						χ^2^	*df*	*p*
		Observed			Expected					
	Sulf2 Sulf1	+/+	+/−	−/−	+/+	+/−	−/−			
*Sulf1* ^+/−^ *Sulf2* ^+/−^×*Sulf1* ^+/−^ *Sulf2* ^+/−^(P9-P10)	+/+	9	33	11	11.125	22.25	11.125	15.865	8	<0.05
	+/−	18	49	18	22.25	44.5	22.25			
	−/−	12	26	**2**	11.125	22.25	**11.125**			
*Sulf1* ^+/−^ *Sulf2* ^−/−^×*Sulf1* ^−/−^ *Sulf2* ^+/−^ (P1-P2)	+/−		31	23		23	23	9.739	3	<0.025
	−/−		27	**11**		23	**23**			
*Sulf1* ^+/−^ *Sulf2* ^−/−^×*Sulf1* ^−/−^ *Sulf2* ^+/−^ (E17.5-E18.5)	+/−		18	20		20.25	20.25	1.617	3	NS
	−/−		25	**18**		20.25	**20.25**			
*Sulf1* ^+/−^ *Sulf2* ^−/−^×*Sulf1* ^−/−^ *Sulf2* ^+/−^(E10.5-E13.5)	+/−		14	19		16.25	16.25	0.908	3	NS
	−/−		17	**15**		16.25	**16.25**			

1 Day of genotyping is indicated in parentheses.

We sought to determine at what stage (embryonic or post-natal) the double mutants were perishing. We first intercrossed *Sulf1* ^+/−^ *Sulf2* ^−/−^ and *Sulf1* ^−/−^ *Sulf2* ^+/−^ mice and isolated embryos at E18.5, which was typically the day of birth in this strain. At E18.5, we observed as many double-mutant embryos as would have been predicted by Mendelian inheritance (∼25%, Table 2). The *Sulf1* ^−/−^ *Sulf2* ^−/−^ offspring, however, had a statistically significant ∼19% decrease in body weight and a ∼12% decrease in crown-rump length (Fig. 2B and data not shown). Neither *Sulf1* ^−/−^ *Sulf2* ^+/−^ nor *Sulf1* ^+/−^ *Sulf2* ^−/−^ single mutants showed any deviation in birth weight from control littermates, thus providing direct evidence for a redundant role for the sulfatases during embryonic development. Although the *Sulf1* ^−/−^ *Sulf2* ^−/−^ embryos were smaller than control littermates, there were no obvious gross changes in morphology. Overt development of the limbs (including digits), head (including palate), tail, and torso all appeared normal. Similar observations of apparently normally developing *Sulf1* ^−/−^ *Sulf2* ^−/−^ embryos were also made in multiple litters at E10.5, E11.5, E12.5, E13.5, and E15.5. A thorough histological examination of sectioned E15.5 embryos revealed no divergence of *Sulf1* ^−/−^ *Sulf2* ^−/−^ from control embryos (data not shown). This indicates that embryonic defects in the double mutants arise late in development.

**Figure 3:**
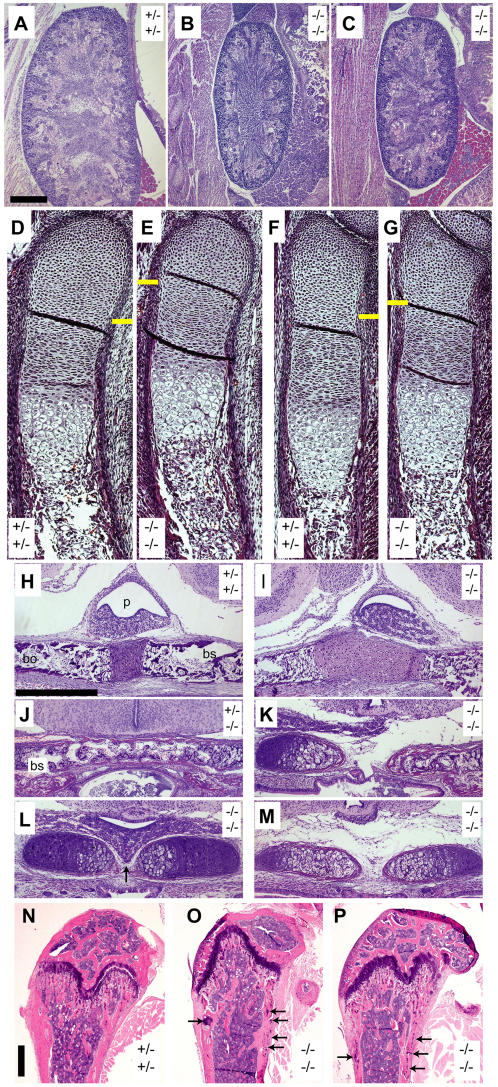
The *Sulf1* ^-/-^ *Sulf2* ^-/-^ mice have multiple subtle anatomic anomalies, including kidney hypoplasia and skeletal defects. (A–C) At E18.5, double knockout fetuses have small kidneys in sagittal sections, and some have attenuated cortices and medullae. (D–G) Sagittal sections of the distal forelimb from E15.5 embryos revealed altered cartilage zonation in the ulna (D, E) and radius (F, G) bones. Yellow lines indicate the transition from distal/low proliferating (above line) to high proliferating (below line) chondrocyte populations as assessed by cellular morphology. (H, I) Sagittal section of the skull base of E18.5 embryos demonstrating abnormal fusion at the cartilaginous interface between the basisphenoid (bs) and basioccipital (bo) bones. (J–M) Coronal section (at the midbrain level) of the skull base at E18.5 showing abnormal midline fusion of the basisphenoid (bs) bone in the double mutant embryos. Bones depicted in panels K, L, and M were from three separate *Sulf1* ^−/−^ *Sulf2* ^−/−^ embryos. Occasionally the pituitary gland descended into the gap at the midline of *Sulf1* ^−/−^ *Sulf2* ^−/−^ embryos (as indicated by the arrow in panel L). (N–P) Representative sections through the proximal tibia of adult females. Arrows indicate regions of retained cartilage in regions that normally consist only of ossified bone. Representative control animals had the *Sulf1* ^+/−^ *Sulf2* ^+/−^ genotype in panels A, D, F, H, and N, and the *Sulf1* ^+/−^ *Sulf2* ^−/−^ genotype in panel J. Representative *Sulf1* ^−/−^ *Sulf2* ^−/−^ double mutant animals are shown in panels B, C, E, G, I, K, L, M, O, and P. Abbreviations used: bo, basioccipital; bs, basisphenoid; p, pituitary gland. The scale bar in (A) represents 500 µm in panels A–C; the scale bar in (H) represents 500 µm in panels H–M; the scale bar in (N) represents 500 µm in panels N–P.

Dissection of E18.5 double mutant embryos showed that all major organs were present and, apart from a slight decrease in size of most organs, appeared normal morphologically. The one clear exception were the kidneys from double mutant neonates, which were decreased in volume by ∼45% relative to kidneys from *Sulf1* ^+/−^ *Sulf2* ^+/−^ control littermates (Fig. 2E). The small kidneys of the *Sulf1* ^−/−^ *Sulf2* ^−/−^ embryos were either normal histologically or, in a few cases, had a modest decrease in the thicknesses of cortex and medulla (compare Fig. 3A with 3B and 3C). All other internal organs examined appeared histologically normal (data not shown).

We then observed the neonates shortly after birth to determine a potential cause of death. Genotyping at postnatal day 1–2, we saw approximately a 50% reduction in expected viability between birth and P1-P2 (Table 2). Of 16 double mutant neonates genotyped at P1-P3, 7 (44%) died before weaning, with most of those dying within the first week of life (data not shown). Of 17 double mutants that survived to weaning, 4 (24%) died within 10 days of weaning. In summary, we observed full viability of *Sulf1* ^−/−^ *Sulf2* ^−/−^ double mutants at E18.5, ∼48% survival at P1-P2, and approximately 18% survival at weaning (Table 4), with roughly a quarter of those pups that survive to weaning dying shortly thereafter (two due to severe malocclusion, others for unknown reasons). The long-term survival frequency of *Sulf1* ^−/−^ *Sulf2* ^−/−^ mice is therefore ∼15%.

**Table 3:** Selected bones of *Sulf1* ^−/−^ *Sulf2* ^−/−^ embryos show decreased volume.

	Humerus	Femur	Frontal	Parietal	Interparietal	Supraoccipital	Basioccipital	Basisphenoid
Control	0.595±0.032	0.431±0.037	0.512±0.017	0.273±0.007	0.171±0.004	0.201±0.013	0.396±0.015	0.295±0.008
Double Mutant	0.468±0.015	0.364±0.010	0.477±0.013	0.263±0.007	0.145±0.008	0.156±0.017	0.335±0.010	0.255±0.008
% decrease	21.4	15.7	6.8	3.7	15.3	22.6	15.5	13.6
*p*	**0.002**	**0.041**	0.152	0.364	**0.042**	0.114	**0.007**	**0.008**

µCT images of E18.5 embryos were thresholded to visualize only mineralized bone. Volumes are expressed in mm^3^ units and are shown as a mean±SEM. The volumes of the left and right humerus, femur, frontal, and parietal bones were averaged to obtain a value for each embryo. Control embryos (*n* = 4) had the *Sulf1* ^+/−^ *Sulf2* ^+/−^ genotype. Double mutant embryos (*n* = 8) had the *Sulf1* ^−/−^ *Sulf2* ^−/−^ genotype. Statistical significance was assessed using a two-tailed Student's *t*-test.

**Table 4:** Penetrance of selected phenotypes in the *Sulf1* ^−/−^ *Sulf2* ^−/−^ animals.

Phenotype	Age	Frequency (%)	*n*
Viability^1^	E10.5-E13.5	92	65
	E17.5-E18.5	89	81
	P1-P2	48	92
	P9-P10	18	178
Bone Morphology^2^			
Sternum defects	E18.5	86	14
Basisphenoid defect	E18.5	100	9
Aberrant BO/BS fusion	E18.5	100	4
Bone volume^3^			
Femur	E18.5	38	8
Humerus	E18.5	88	8
Interparietal	E18.5	75	8
Supraoccipital	E18.5	75	8
Basioccipital	E18.5	75	8
Basisphenoid	E18.5	100	8
Kidney volume^3^			
Adult	14–15 w	80	5
Embryo	E18.5	100	3

Refer to text for complete description of phenotypes observed. Abbreviations used: BO, basioccipital; BS, basisphenoid; w, weeks.

1 Frequency defined as the number expected (by standard Mendelian segregation, see Table 2) divided by the number observed; *n* defined as total number of animals analyzed in a given age range

2 Frequency defined as the percentage of *Sulf1* ^−/−^ *Sulf2* ^−/−^ embryos that showed the described phenotype; *n* was the number of *Sulf1* ^−/−^ *Sulf2* ^−/−^ embryos analyzed

3 Frequency defined as the percentage of *Sulf1* ^−/−^ *Sulf2* ^−/−^ animals whose mean volume for the kidney or indicated bone was less than the smallest of the control animals' kidney or bone volumes; *n* as the number of *Sulf1* ^−/−^ *Sulf2* ^−/−^ animals analyzed

Consistent with the observed low birth weight, those double mutants surviving to P1-2 showed ∼25% reduction in body weight (data not shown). Although the *Sulf1* ^−/−^ *Sulf2* ^−/−^ neonates had a suitable skin color (pink, indicating sufficient oxygenation) and apparently normal motility, they frequently did not have milk in their stomachs, suggesting that the cause of early neonatal death may have been related to undernourishment or dehydration. Histological analysis of placenta, kidney, stomach, intestine, pancreas, liver, and heart from double mutant neonates revealed no obvious abnormalities explaining the inability to suckle (data not shown). Thus, the cause of neonatal mortality remains undetermined. However, a likely premise is that double knockout neonates had neurological problems (suggested by the extensive *Sulf1* and *Sulf2* gene expression patterns in the embryonic central nervous system). More thorough neuroanatomic and neurobehavioral assays are ongoing to further investigate the cause of neonatal lethality.

### Surviving *Sulf1* ^−/−^ *Sulf2* ^−/−^ adults are small, exhibit subtle skeletal and renal defects, but are otherwise normal in gross and histological structure

We were able to analyze a total of 10 adult (5 male and 5 female) *Sulf1* ^−/−^ *Sulf2* ^−/−^ animals. All animals showed below-average weight, with one representative cohort of ∼14–15 week-old double mutant males (*n* = 5) having an average weight 26% less than age-matched littermate control males (*n* = 10) (Fig. 2D). Another cohort of three *Sulf1* ^−/−^ *Sulf2* ^−/−^ and two control females (matched at 11 weeks of age) also exhibited a decreased body weight, with double mutants weighing 29% less than controls (13.8 *g vs*. 19.5 *g*, respectively; data not shown).

We collected and weighed a full panel of organs from the adult *Sulf1* ^−/−^ *Sulf2* ^−/−^ and control mice. When corrected for the aforementioned decrease in body weight, all the organs appeared to be of generally normal size and gross appearance except for the kidneys, which were disproportionately small. For instance, in the male cohort described above, the kidneys of double mutant animals showed a 44% decrease in weight relative to controls (Fig. 2F), which is substantially greater than the 26% discrepancy in total body weight. Despite the decreased kidney size, renal function did not appear to be altered in the double mutants, with normal levels of serum and urine markers testing kidney function (data not shown). This is consistent with the observation that the kidneys of double mutant adults were histologically normal (data not shown).

Whereas the *Sulf1* ^−/−^ single mutant mice were similar in body and kidney weight to age-matched control adult males, *Sulf2* ^−/−^ mice, like the *Sulf1*; *Sulf2* double mutants, had decreased body weight (20% decrease) and kidney weight (29% decrease). The degree of decrease in relative kidney and body weight was less in the *Sulf2* single mutants than in the *Sulf1*; *Sulf2* double mutants, indicating that although *Sulf2* appears to be the more important of the two sulfatase genes in regulating kidney development, *Sulf1* also contributes to kidney growth. Therefore, *Sulf1* and *Sulf2* appear to play redundant roles in the regulation of body and kidney weight.

Histological analysis of a panel of organs collected from the adult double mutant animals revealed all tissues examined to be histologically normal, with two exceptions. First, one 11-week-old double mutant female had interstitial pneumonia with syncytial cell formation localized to the peribronchiolar region, suggesting the possibility of a latent phenotype related to immunological dysfunction. A survey of immunological cell types in the peripheral blood and spleen showed no obvious defect in immune cell development of the double mutant adults (data not shown). However, more specific analyses might yet reveal a role of the sulfatases in immune function. Second, all of the *Sulf1* ^−/−^ *Sulf2* ^−/−^ adults examined showed retention of cartilage cores in the cortical bone of the tibial metaphysis, sometimes in conjunction with trabecular disorganization (compare Fig. 3N with 3O and 3P). In summary, surviving *Sulf1* ^−/−^ *Sulf2* ^−/−^ adults are small in stature, but show apparently normal behavior and have grossly and histologically normal tissues, with the lone exception of the subtle changes observed in the bones.

### *Sulf1* ^−/−^ *Sulf2* ^−/−^ embryos show defects in bone development

**Figure 4:**
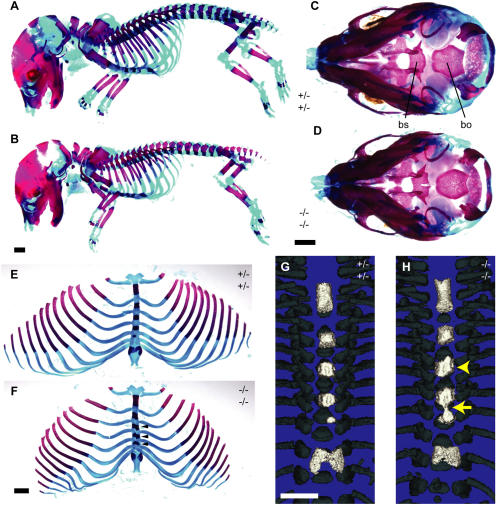
Embryos deficient for both *Sulf1* and *Sulf2* had several distinct defects in bone development. (A–F) Representative images of E18.5 littermate control *Sulf1* ^+/−^ *Sulf2* ^+/−^ (A, C, E, G) and *Sulf1* ^−/−^ *Sulf2* ^−/−^ (B, D, F, H) fetuses double-stained with alcian blue and alizarin red to demonstrate skeletal elements. (A, B) Whole-mount images demonstrating the reduced size of the double mutant skeleton. All bones are present, and no obvious fusion defects are apparent in any class of joints. (C, D) Skulls viewed from the posterior side. Note the hole in the dorsal side of the basisphenoid bone of the *Sulf1* ^-/−^ *Sulf2* ^−/−^ skeleton (to the right in D). Also note that the palate appears to be developing properly. (E–H) Rib cages (skeletal double staining; E, F) and sternums (thresholded µCT; G,H) revealing sternebral asymmetry about the midline in double knockout mice (black arrowheads in F, and yellow arrowhead in H). The 4^th^ and 5^th^ sternebrae (counting from rostral to caudal) have a slender site of ossification bridging them at the midline (F, with another example in panel H [yellow arrow]). Scale bars in panels B, D, F, and G represent 1 mm for panels A/B, C/D, E/F, and G/H, respectively.

Given our observation of a bone defect in the *Sulf1* ^−/−^ *Sulf2* ^−/−^ adults (Fig. 3O, P) and the expression of *Sulf1* and *Sulf2* at several sites in bone during embryogenesis (Fig. S2 and S4 and data not shown), we analyzed bone development in the *Sulf1* ^−/−^ *Sulf2* ^−/−^ embryos. To this end, we stained dehydrated E18.5 embryos with alcian blue and alizarin red to assess bone and cartilage formation. In general, the skeletons of double mutants were similar to those of control littermates, though notably smaller in stature (Fig. 4A, B). One highly penetrant difference (12 of 14 (86%) *Sulf1* ^−/−^ *Sulf2* ^−/−^ E18.5 embryos examined) was the asymmetry and/or fusion of sternebrae (Fig. 4E–H, Table 4). Only 1 of 19 (∼5%) control embryos showed this anomaly. High penetrance of this defect required both *Sulf1* and *Sulf2* deficiency, in that only one of five *Sulf1* ^−/−^ and none of three *Sulf2* ^−/−^ embryos exhibited sternebral defects (data not shown).

Histological examination of E18.5 embryos focusing on skeletal development revealed two other subtle but highly penetrant alterations in *Sulf1*- and *Sulf2*-deficient embryos. These changes included (i) non-fusion of the posterior basisphenoid bone (Figs. 3K, L, and M; Fig. 4D; Table 4), in one case with the pituitary gland extending ventrally into the gap (arrow, Fig. 4L); and (ii) aberrant fusion of the basioccipital and basisphenoid bones indicated by widening and bulging of the cartilage interface between these two bones (Fig. 3I, Table 4). The increased prevalence of each of these lesions in *Sulf1* ^−/−^ *Sulf2* ^−/−^ embryos as compared to control embryos was statistically significant (*p*<0.02 by Chi square method) using blinded analysis or, when appropriate, direct slide-by-slide comparison.

Seeking a more quantitative analysis of the skeletal phenotype of the *Sulf1* ^−/−^ *Sulf2* ^−/−^ embryos, we analyzed E18.5 embryonic skeletons by micro-computed tomography (µCT). Assessing the ossified bone volume of several different bones in the skeleton, we found that the double mutants exhibited a decrease in bone volume (Tables 3 and 4) and length (data not shown) of the humerus and femur. Likewise, the interparietal, basisphenoid, basioccipital, and supraoccipital bones of the skull also had decreased volumes (usually ∼15% smaller than bones from control embryos, Table 3), though the decreased volume of the supraoccipital bone was observed with only 50% penetrance (4 of 8 embryos analyzed had a mean volume of less than 2 SD below the mean of age-matched controls). In contrast, the volume of the frontal and parietal bones of the skull showed no difference between double mutants and controls. In summary, all bones examined except the frontal and parietal bones of the skull were ∼15–20% smaller in the *Sulf1* ^−/−^ *Sulf2* ^−/−^ embryos as compared to *Sulf1* ^+/−^ *Sulf2* ^+/−^ littermate controls (Table 3). Interestingly, none of the bones from *Sulf1* ^−/−^ *Sulf2* ^−/−^ embryos showed a change in average bone density as compared to control littermates (data not shown), indicating that the ossified regions of the double mutant embryos' bones were smaller but not less dense.

Seeking an explanation for the shorter long bones with decreased volume, we assessed the bones of E15.5 embryos. *Sulf1* ^−/−^ *Sulf2* ^−/−^ embryos showed slightly shorter long bones in all limbs. All expected chondrocyte cell populations (distal, proliferative, and hypertrophic) appeared to be present (Fig. 3D–G). Overt chondrocyte differentiation was not altered, in that we observed normal expression patterns of *Collagen II* and *Collagen X* (Fig. S6). One apparent defect in the double mutants was a smaller distal (low proliferating) chondrocyte population (compare panels D with E and F with G in Fig. 3). In contrast, both the proliferative and hypertrophic chondrocyte populations appeared to be present in comparable numbers.

### *Sulf1* ^−/−^ *Sulf2* ^−/−^ cells are hyper-responsive to fibroblast growth factors (FGF)

Previous work has shown that the sulfation status of heparan sulfate is important for its promotion of FGF signaling [27]–[29]. Over-expression of Sulf1 in tumor cell lines acts to inhibit FGF-dependent signaling [23]. Furthermore, transgenic mice engineered to express a hyperactive mutant allele of FGFR1 [30] or FGFR3 [31] in bone results in sternal fusion, a phenotype we observed in our sulfatase-deficient embryos.

**Figure 5:**
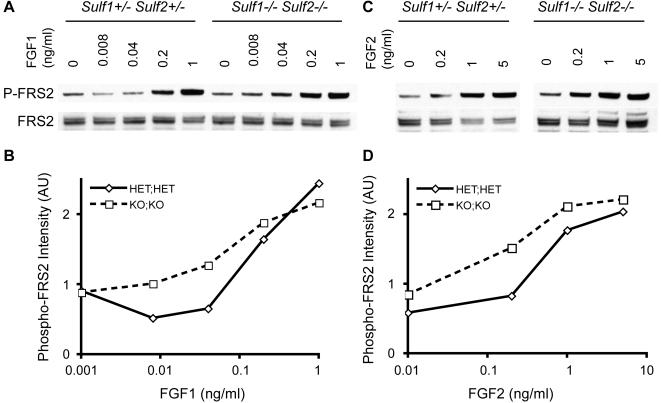
*Sulf1* ^−/−^ *Sulf2* ^−/−^ embryonic fibroblasts are hyper-sensitive to fibroblast growth factors. Fibroblasts were stimulated with differing doses of FGF1 (A, B) or FGF2 (C, D) for 10 minutes, then assessed for activation of signaling by Western analysis of phosphorylated FRS2. Results are representative of three independent experiments.

We therefore sought to assess the FGF sensitivity of cells from *Sulf1* ^−/−^ *Sulf2* ^−/−^ embryos. To accomplish this, we derived fibroblast cell cultures from embryos and assessed their responsiveness to different doses of FGF1 and FGF2, using phosphorylation of the FGFR signaling adaptor FRS2 as a marker of pathway activation [32]. Whereas *Sulf1* ^−/−^ *Sulf2* ^−/−^ and *Sulf1* ^+/−^ *Sulf2* ^+/−^ control MEFs showed similar kinetics of FRS2 phosphorylation when assessed at a saturating concentration of FGF1 and FGF2 (data not shown), sulfatase-deficient MEFs were hyper-sensitive to both FGF1 and FGF2, activating half-maximal FRS2 phosphorylation at ∼5-fold lower concentration of FGFs (Fig. 5). This demonstrates that the secreted sulfatases are involved with modulation of FGF signaling and suggests a potential mechanism for the observed sternal fusion phenotype.

## Discussion

### *Sulf1* and *Sulf2* perform redundant and essential functions during embryonic and neonatal development

We describe here the phenotype of mice engineered to contain retroviral gene trap alleles inserted into the *Sulf1* and/or *Sulf2* loci. We showed that the *Sulf1* and *Sulf2* gene trap alleles are profoundly hypomorphic, with no compensatory changes in paralog gene expression (Fig. 1). Mice deficient for either *Sulf1* or *Sulf2* alone were viable, fertile, and normal by nearly all criteria examined (Table 1, Fig. 2). *Sulf2*-deficient adult mice did show a tendency towards decreased body weight relative to control animals, but this tendency was not accompanied by any obvious functional or structural changes. In contrast to a recent report [19], we observed no deviation from the expected Mendelian birth ratios of homozygous *Sulf2* mutant animals, either in our original mixed 129/SvEvBrd; C57BL/6 background or in mice at the N4 generation of backcrossing into the C57BL/6N strain (Table 1). We also did not observe the previously described runting or lung defects (Fig. 2 and data not shown)[19]. Perhaps the discrepancies between our results and those of Lum *et al*. [19] were due to the difference in the 129 genetic strain background used (129/SvEvBrd in this study *vs*. 129P2/Ola in Lum *et al*.): the previously-reported reduction in decreased *Sulf2* ^−/−^ viability was only seen in the 129P2/Ola; C57BL/6 mixed background and not in two other more homogeneous backgrounds (C57BL/6 [N3 and N7] and FVB/N [N1 and N7]). Alternatively, the degree of hypomorphism and/or the nature of any aberrant transcripts generated by the different gene trap alleles used in our and the previous study might have affected the phenotypic outcome. In either case, the effect(s) of *Sulf2* deficiency are clearly quite subtle.

We also showed that, as remarked upon briefly by Lamanna *et al*. [17], [18], *Sulf1* and *Sulf2* are functionally redundant genes essential for viability. We report here the first characterization of the penetrance and timing of perinatal lethality in mice deficient for *Sulf1* and *Sulf2*, as well as the phenotype of the double mutant embryos and neonates. Though *Sulf1* and *Sulf2* are expressed in a wide variety of embryonic and adult tissues (Fig. 1; Figs. S1, S2, S3, S4, S5), the development of secreted sulfatase-deficient mice proceeds with remarkably normal integrity, with the sole developmental alterations observed to be decreased body size accompanied by subtle kidney and bone defects. The functions of the secreted sulfatases, though obviously important as evidenced by the neonatal lethality in their absence, are likely to be understated, perhaps via refining or restricting the extent of some other essential signaling pathways.

The bone defects observed in the *Sulf1* ^−/−^ *Sulf2* ^−/−^ embryos are remarkable in several ways. First, there does appear to be a deficit in bone growth in the double mutants, in that bones located in both the axial and appendicular skeleton, which form by the distinct processes of endochondral and intramembranous ossification, showed a decrease in ossified bone volume (Tables 3 and 4). Second, although the secreted sulfatase-deficient skeletons appeared overall to be patterned normally (Fig. 4A and B), we did observe several highly specific morphological defects at the midline, including sternal fusion and defective basisphenoid patterning. Interestingly, sternal defects such as those exhibited by the *Sulf1* ^−/−^ *Sulf2* ^−/−^ embryos have been reported not only in the *Hs2st*-deficient mice (see below), but also in mice with compound mutations in different *Bmp* family members [33], *EphrinB1* mutants [34], and in a mice engineered to contain extra copies of a hypermorphic *Fgfr1* allele or transgenic expression of hypermorphic *Fgfr3* allele [30], [31]. Perhaps this convergence in phenotypes indicates a role for the secreted sulfatases in the regulation of Bmp, Fgf, and/or Ephrin signaling during sternal development. Our demonstration that *Sulf1* ^−/−^ *Sulf2* ^−/−^ fibroblasts are hypersensitive to FGF signaling (Fig. 5) supports this supposition. Third, whereas nearly all joints examined in the *Sulf1* ^−/−^ *Sulf2* ^−/−^ double mutants appeared grossly and histologically normal (Fig. 4), two joints in the base of the skull had notable, yet distinct defects (a thickening of the basioccipital/basisphenoid suture and a failure of the posterior basisphenoid bone to fuse at the midline). A similar basisphenoid defect has been reported in *Bmp7*- and *Cdon*-null embryos [35], [36]. This phenocopy congruence supports the possible role of secreted sulfatases in regulating Bmp and Hedgehog signaling. Finally, it is worth noting several phenotypes that were *not* observed in the *Sulf1* ^−/−^ *Sulf2* ^−/−^ embryos; in particular, we did not find skull vault suture defects, cleft palate, appendicular joint fusions, or polydactyly. All of these defects have been associated with altered Fgf, Bmp, or Hh signaling. An interesting implication of this discrepancy is that the sulfatases might regulate heparan sulfate-dependent growth factor signaling at some but not all developing bone sites. Further work will be required to confirm this possibility.

The exact cause of neonatal lethality remains unclear. Even if the observed decrease in kidney size were to result in altered renal function (a prediction not supported by the apparently normal kidney function we measured in the *Sulf1* ^−/−^ *Sulf2* ^−/−^ adults that had disproportionately small kidneys), one would not expect such a precipitous decline in neonatal survival. The normal structure of thoracic viscera and their branches (data not shown) suggested that altered cardiovascular or pulmonary function–a common etiology for perinatal mortality–was also not the cause of lethality in this case. Likewise, the digestive organs and skeleton did not exhibit substantive anatomic abnormalities (data not shown). The frequent absence of milk spots in double knockout neonates suggested that an absence or reduction in suckling, leading ultimately to dehydration, might have been a cause of mortality in the double mutants. A potentially deficient feeding response might in turn be due to underlying neurological defects, as suggested by examination of other genes expressed in the central nervous system [37], [38]. We are currently exploring this possible pathogenesis as an explanation for the lethality observed in *Sulf1* ^−/−^ *Sulf2* ^−/−^ neonates.

### The phenotype of the surviving *Sulf1* ^−/−^ *Sulf2* ^−/−^ mice is similar to that of *Hs2st* mutant mice

Several recent studies have revealed an unexpected compensatory link between the 6-*O* and 2-*O* sulfation states of heparan sulfate, in both vertebrate (mouse) and invertebrate (*Drosophila*) systems [17], [39], [40]. Specifically, tissues from mice with a gene trap disruption of the single 2-*O*-sulfotransferase (*Hs2st*) gene exhibit not only the expected decrease in 2-*O*-sulfated iduronic acid, but also an apparently compensatory increase in 6-*O* sulfation of *N*-sulfoglucosamine [40]. Lamanna *et al*. recently reported that embryonic fibroblasts derived from mice devoid of both the *Sulf1* and *Sulf2* secreted sulfatases had not only the expected increase in 6-*O* sulfation of glucosamine, but also a decrease in 2-*O* sulfated iduronic acid [17], the same trends as reported for the *Hs2st*-deficient mice.

Mice deficient for *Hs2st* exhibit defective skeletal development, bilateral kidney agenesis, cleft palate, decreased cerebral cortex thickness, polydactyly, and iris coloboma. In addition, *Hs2st*-deficent mice are perinatal lethal with full penetrance [9], [41]. The *Sulf1*; *Sulf2*-deficient mice, as reported here, showed phenotypic similarity to the *Hs2st*-deficient mice, albeit of lesser severity. Specifically, both mutant strains of mice exhibited sternal fusions, defects in kidney development, and perinatal lethality, indicating that *Hs2st* and the secreted sulfatases might regulate similar developmental processes. In contrast, *Sulf1* ^−/−^ *Sulf2* ^−/−^ mice did not show cleft palate, cerebral cortex diminution, digit duplications, or eye defects, despite the fact that *Sulf1* and *Sulf2* are expressed in all of these tissues (Figs. S2 and S4 and data not shown). The differences between the phenotypes of the two mice might indicate (i) that the sulfatases are not expressed at the correct time and/or place to play a role in all of the same developmental processes as *Hs2st*, (ii) that the sulfation status changes of the sulfotransferase- and sulfatase-deficient tissues differ in some way not revealed by the previous studies, or (iii) that the effect of deficiency of an intracellular biosynthetic enzyme, *Hs2st*, might be more profound than the deficiency of secreted enzymes, *Sulf1* and *Sulf2*, that act *ex post facto*.

### The subtlety of the *Sulf1; Sulf2* double deficient phenotype would not have been predicted based upon previous *in vitro* experiments

Based almost entirely upon over-expression studies in cell lines grown *in vitro*, the secreted sulfatases have been shown to modulate a wide variety of heparin-binding growth factor signaling pathways, including WNT, FGF, HB-EGF, HGF, and BMP [16], [23], [42]–[45]. Except for the cases of WNT1 and BMP4, wherein elevated expression of the sulfatases promotes cell signaling, the literature is in agreement that forced (over)expression of *Sulf1* strongly inhibits signaling by various heparin-binding growth factors (including FGF2, HB-EGF, and HGF). Furthermore, recombinant Sulf2 enzyme has been shown to release heparan sulfate- and heparin-bound signaling proteins such as VEGF_165_, FGF1, and SDF1 [46], conferring another potential set of non-cell autonomous functions to the secreted sulfatases.

Given that most of the aforementioned signaling pathways are critical for certain developmental processes and patterning events, one might have predicted from the *in vitro* studies that secreted sulfatase-deficient mice would have exhibited profound developmental defects in a multitude of organ systems. This prediction was not borne out by the present organismal loss-of-function study. On the contrary, we show here that mice with disrupted *Sulf1* and *Sulf2* genes are by and large normal, with a few subtle patterning and growth defects. As evidenced by the high penetrance neonatal lethality, clearly the secreted sulfatases are essential for some process critical for life. Perhaps these enzymes are involved in fine-tuning rather than directing the spatial distribution of heparan sulfate-binding factors.

### Conclusion

In summary, we have shown that the *Sulf1* and *Sulf2* secreted 6-*O*-endosulfatase genes are redundant and essential for post-natal survival in developing mice. Whereas animals deficient for either *Sulf1* or *Sulf2* alone have normal birth weight, full viability, and histologically normal organs, mice simultaneously deficient for both *Sulf1* and *Sulf2* exhibit low birth weight, ∼50% neonatal lethality, ∼80% lethality as of weaning, failure to thrive, and a variety of skeletal and renal defects that arise during late embryogenesis.

## Supporting Information

Figure S1 Loss of Sulf1 protein expression in *Sulf1* ^−/−^ embryos. Immunohistochemistry using a Sulf1-specific affinity purified polyclonal antibody was performed on frozen sections of WT (A, C) or *Sulf1* ^−/−^ (B, D) embryos. Note the loss of Sulf1 expression in the floorplates of *Sulf1* ^−/−^ embryos. Floor plate cells are outlined in yellow to facilitate visual comparison.
(5.07 MB TIF)

Figure S2 *Sulf1* is expressed in a dynamically shifting pattern throughout mouse embryonic development. Whole mount in situ hybridization (ISH) using a DIG-labeled *Sulf1* probe was performed on WT embryos at ages E9.5 (A, F), E10.5 (B, G), and E11.5 (C, H). Embryos in (F–H) are viewed from a rostral position, with dorsal at the top. Section-based ISH using ^35^S-labeled *Sulf1* probe was performed on 20-μm transverse forelimb-level sections of E10.5 (D, E), E11.5 (I–J), and E12.5 (K–N) embryos. Abbreviations used: ao, aorta; ar, artery; b, bronchus; cp, choroid plexus; cv, cardinal vein; e, esophagus; lb, distal forelimb bud; n, notochord; nt, neural tube; p, palate; sc, condensing sclerotome; t, tongue. Scale bar in (A) corresponds to 1 mm in panels A, B, and C, and 0.67 mm in panels F, G, and H. Scale bar in (J) represents 200 μm in panels D, E, I, J, K, L, M, and N.
(3.64 MB TIF)

Figure S3 *Sulf1* is expressed in many tissues of adult mice. Expression of *Sulf1* in tissues derived from wild-type C57BL/6 adults, as determined by microarray analysis (Gene Logic, Inc., Gaithersburg, MD). MAS5.0 values are shown for probeset 113914_at on the MGU74B Affymetrix chip (Affymetrix, Santa Clara, CA). Each point refers to a different sample; the lines represent the mean expression value for each tissue. Similar expression patterns were also observed using probeset 116019_at and in tissues derived from 129 and DBA strains of mice (data not shown).
(0.45 MB TIF)

Figure S4 *Sulf2* is expressed abundantly and dynamically at many locations in developing mouse embryos. β-Galactosidase activity (from the VICTR37 gene trap allele inserted in the *Sulf2* locus) was detected in whole mount preparations using a standard lacZ histochemistry method at E10.5 (A), E11.5 (B, C), and E12.5 (D, E). (A) The degree of staining corresponds to the number of gene trap alleles inherited, with WT animals (upper) showing undetectable X-gal deposition and homozygous embryos (bottom) showing more intense staining than heterozygous embryos (middle). *Sulf2* ^+/−^ (B, C) and *Sulf2* ^−/−^ (D, E) embryos were imaged after detection of β-galactosidase activity. Section-based in situ hybridization using ^35^S-labeled *Sulf2* probe was performed on 20-μm transverse forelimb-level sections of E11.5 (F–G) and E12.5 (H–K) embryos. The same abbreviations were used as in Figure 2. Scale bar in (A) corresponds to 1 mm in panels A, B, C, and E. The scale bar in (D) is 1 mm. The scale bar in (G) represents 200 μm in panels F–K.
(3.52 MB TIF)

Figure S5 *Sulf2* is also expressed in numerous tissues in adult and developing mice. Expression of *Sulf2* in tissues derived from duplicate wild-type C57BL/6 adults (upper two panels) and embryonic samples (lower panel), as determined by consulting the microarray analysis submitted to Gene Expression Omnibus (GEO) with accession number GSE1133. Expression values are shown for probeset gnflm29631_a_at on the custom Affymetrix chip [25]. Each point refers to a different sample; the lines represent the mean expression value for each tissue.
(0.61 MB TIF)

Figure S6 *In situ* hybridization of bone differentiation markers in sulfatase-deficient embryos. *Collagen II* (A, B) and *Collagen X* (C, D) expression were assessed in sections of distal ulnas of control *Sulf1* ^+/−^ *Sulf2* ^+/−^ (A, C) and *Sulf1* ^−/−^ *Sulf2* ^−/−^ (B, D) E15.5 embryos.
(10.15 MB TIF)
