# Supplementary figures and images for: Secreted Sulfatases Sulf1 and Sulf2 Have Overlapping yet Essential Roles in Mouse Neonatal Survival
Source: PLoS One. 2007 Jun 27;2(6):e575. doi: 10.1371/journal.pone.0000575 (PMC1892809; doi:10.1371/journal.pone.0000575)

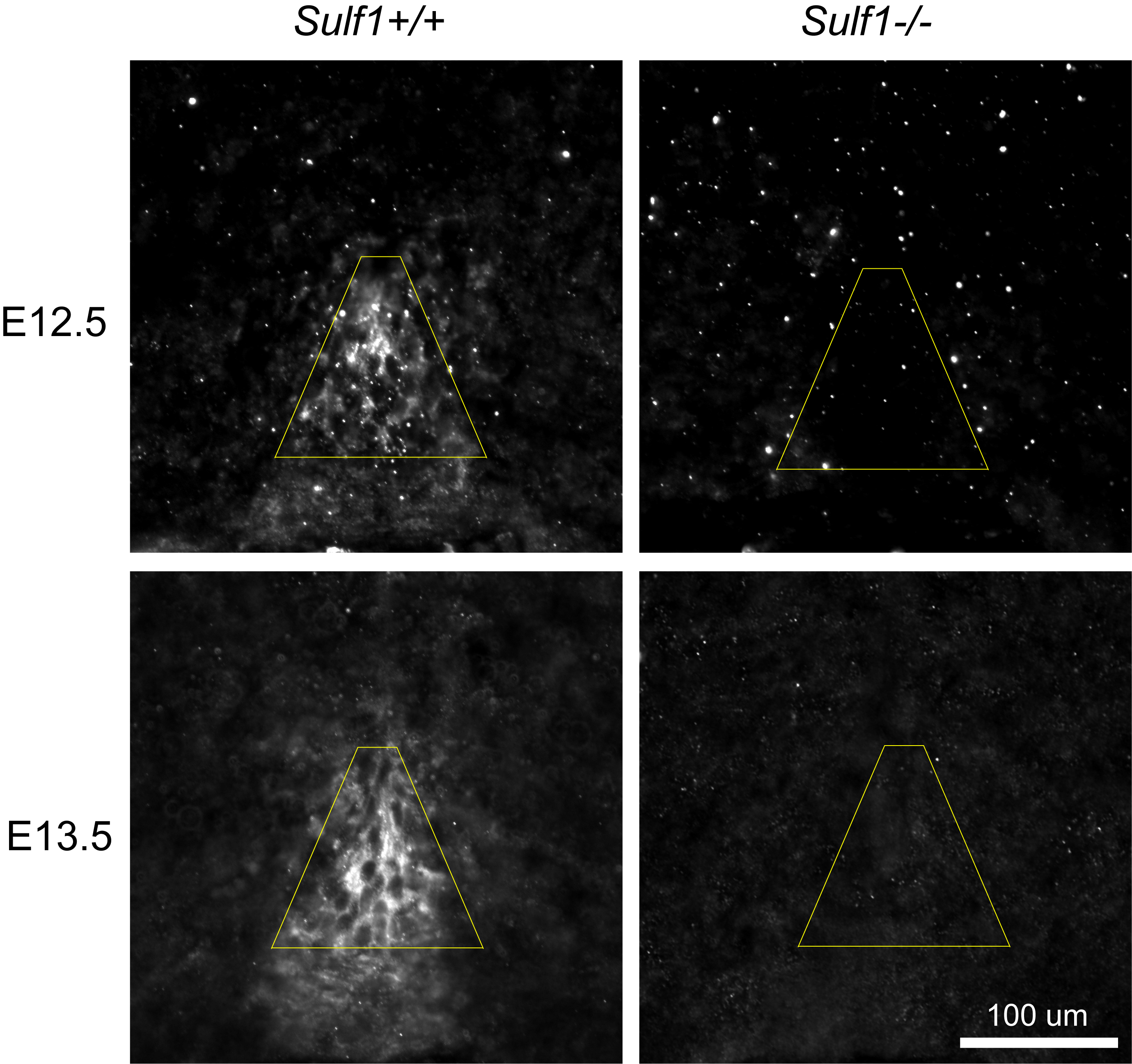

Supplement: Figure S1 — Loss of Sulf1 protein expression in Sulf1 −/− embryos. Immunohistochemistry using a Sulf1-specific affinity purified polyclonal antibody was performed on frozen sections of WT (A, C) or Sulf1 −/− (B, D) embryos. Note the loss of Sulf1 expression in the floorplates of Sulf1 −/− embryos. Floor plate cells are outlined in yellow to facilitate visual comparison. (5.07 MB TIF) [file pone.0000575.s001.tif]

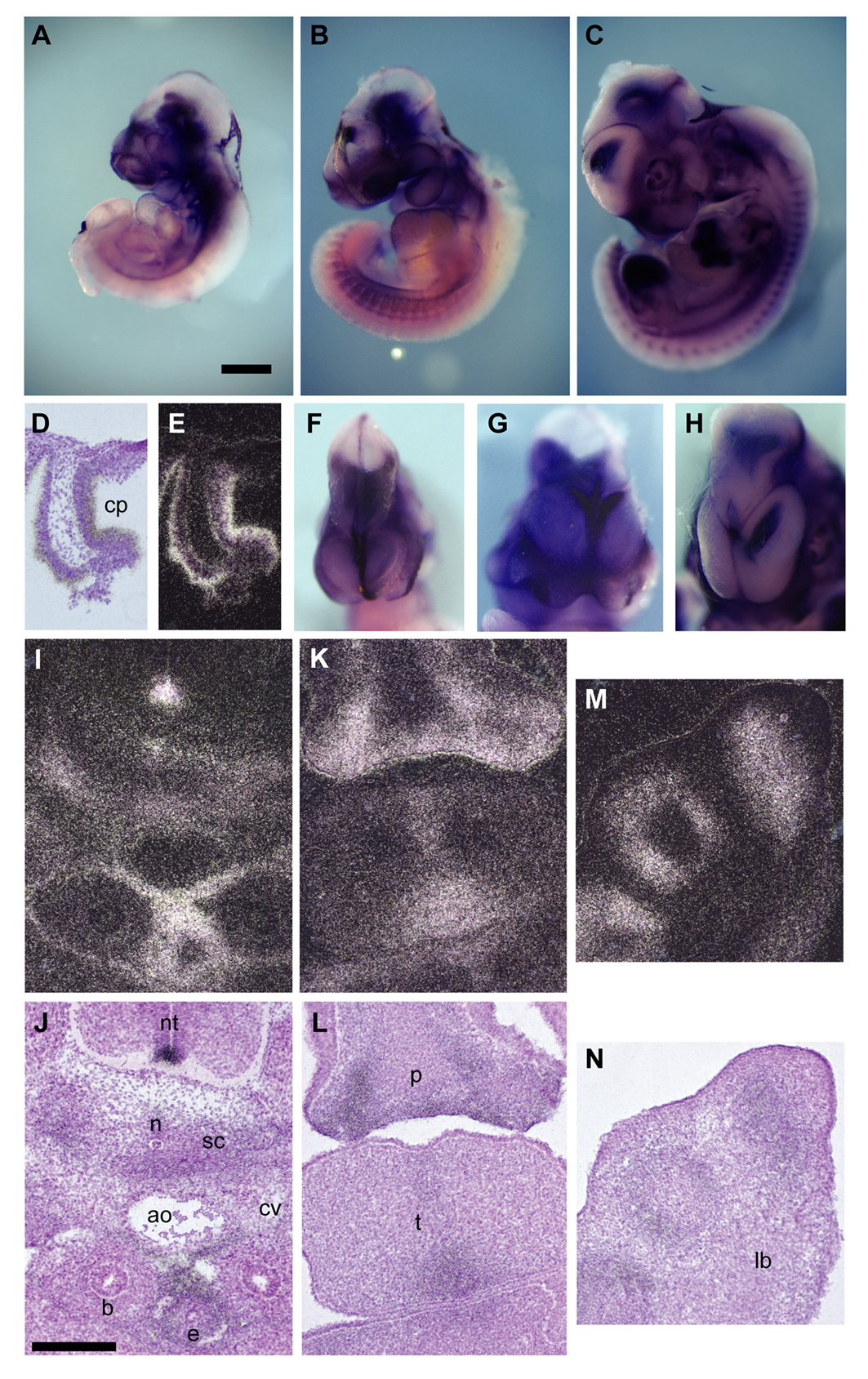

Supplement: Figure S2 — Sulf1 is expressed in a dynamically shifting pattern throughout mouse embryonic development. Whole mount in situ hybridization (ISH) using a DIG-labeled Sulf1 probe was performed on WT embryos at ages E9.5 (A, F), E10.5 (B, G), and E11.5 (C, H). Embryos in (F–H) are viewed from a rostral position, with dorsal at the top. Section-based ISH using 35S-labeled Sulf1 probe was performed on 20-μm transverse forelimb-level sections of E10.5 (D, E), E11.5 (I–J), and E12.5 (K–N) embryos. Abbreviations used: ao, aorta; ar, artery; b, bronchus; cp, choroid plexus; cv, cardinal vein; e, esophagus; lb, distal forelimb bud; n, notochord; nt, neural tube; p, palate; sc, condensing sclerotome; t, tongue. Scale bar in (A) corresponds to 1 mm in panels A, B, and C, and 0.67 mm in panels F, G, and H. Scale bar in (J) represents 200 μm in panels D, E, I, J, K, L, M, and N. (3.64 MB TIF) [file pone.0000575.s002.tif]

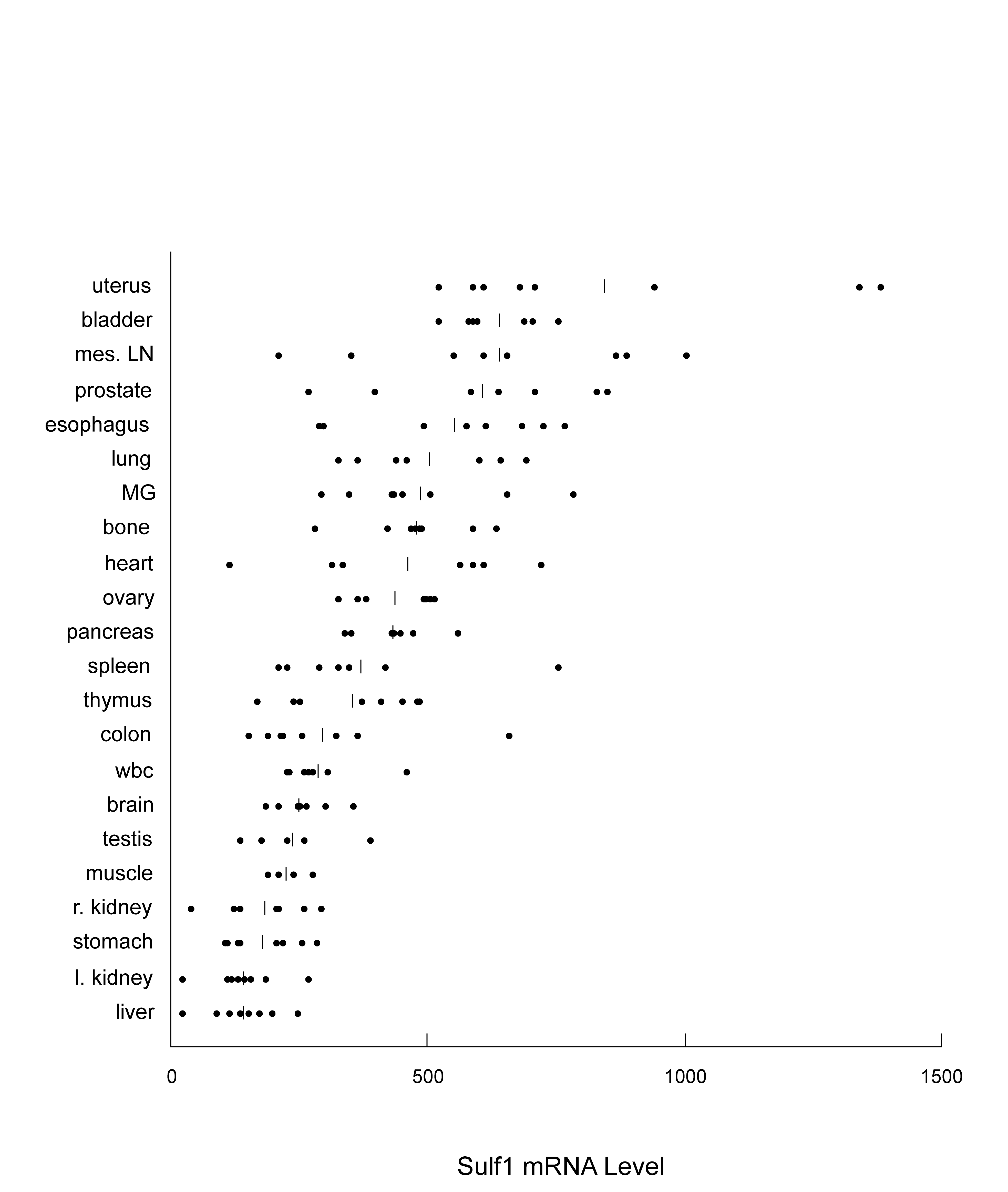

Supplement: Figure S3 — Sulf1 is expressed in many tissues of adult mice. Expression of Sulf1 in tissues derived from wild-type C57BL/6 adults, as determined by microarray analysis (Gene Logic, Inc., Gaithersburg, MD). MAS5.0 values are shown for probeset 113914_at on the MGU74B Affymetrix chip (Affymetrix, Santa Clara, CA). Each point refers to a different sample; the lines represent the mean expression value for each tissue. Similar expression patterns were also observed using probeset 116019_at and in tissues derived from 129 and DBA strains of mice (data not shown). (0.45 MB TIF) [file pone.0000575.s003.tif]

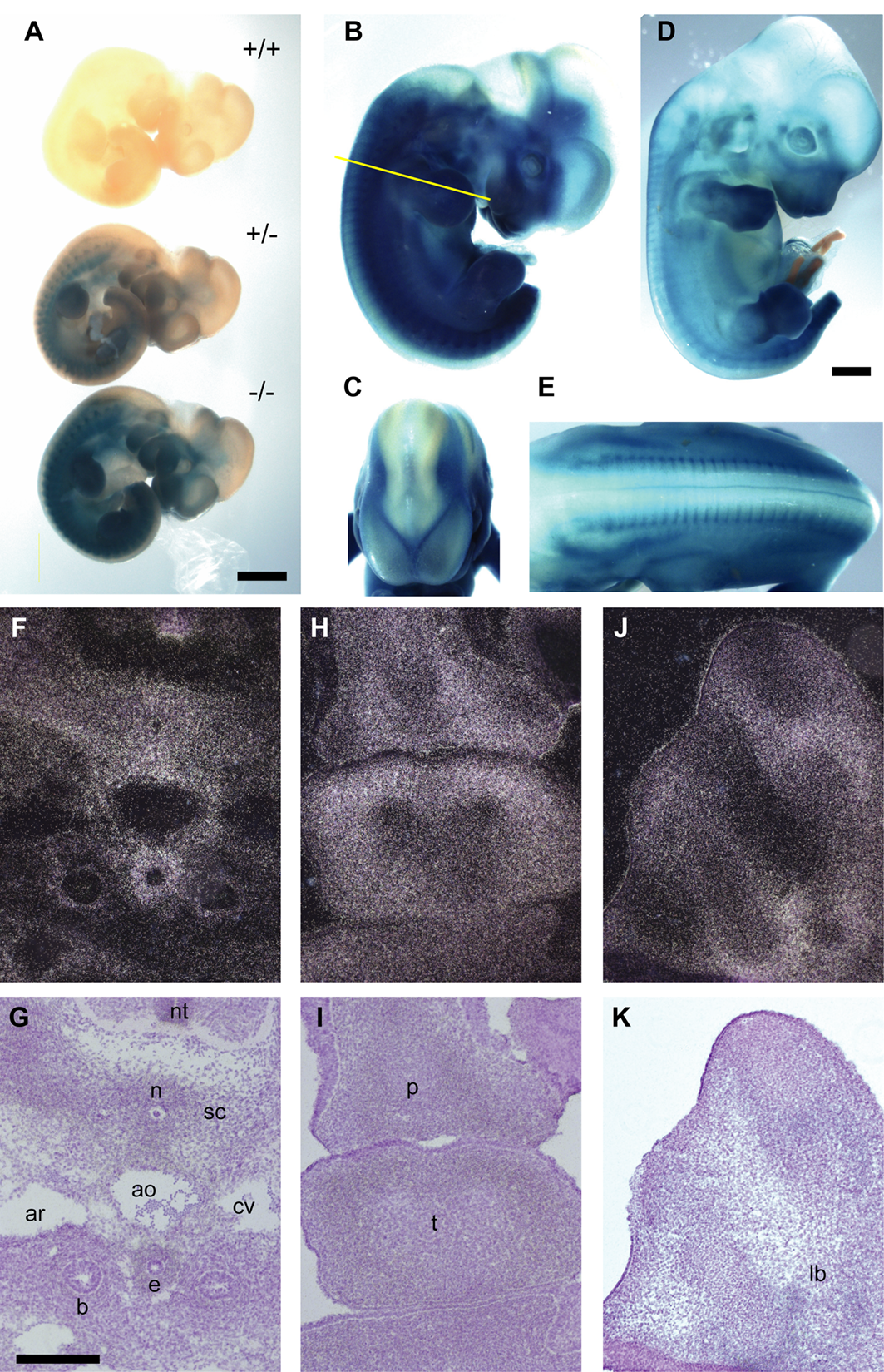

Supplement: Figure S4 — Sulf2 is expressed abundantly and dynamically at many locations in developing mouse embryos. β-Galactosidase activity (from the VICTR37 gene trap allele inserted in the Sulf2 locus) was detected in whole mount preparations using a standard lacZ histochemistry method at E10.5 (A), E11.5 (B, C), and E12.5 (D, E). (A) The degree of staining corresponds to the number of gene trap alleles inherited, with WT animals (upper) showing undetectable X-gal deposition and homozygous embryos (bottom) showing more intense staining than heterozygous embryos (middle). Sulf2 +/− (B, C) and Sulf2 −/− (D, E) embryos were imaged after detection of β-galactosidase activity. Section-based in situ hybridization using 35S-labeled Sulf2 probe was performed on 20-μm transverse forelimb-level sections of E11.5 (F–G) and E12.5 (H–K) embryos. The same abbreviations were used as in Figure 2. Scale bar in (A) corresponds to 1 mm in panels A, B, C, and E. The scale bar in (D) is 1 mm. The scale bar in (G) represents 200 μm in panels F–K. (3.52 MB TIF) [file pone.0000575.s004.tif]

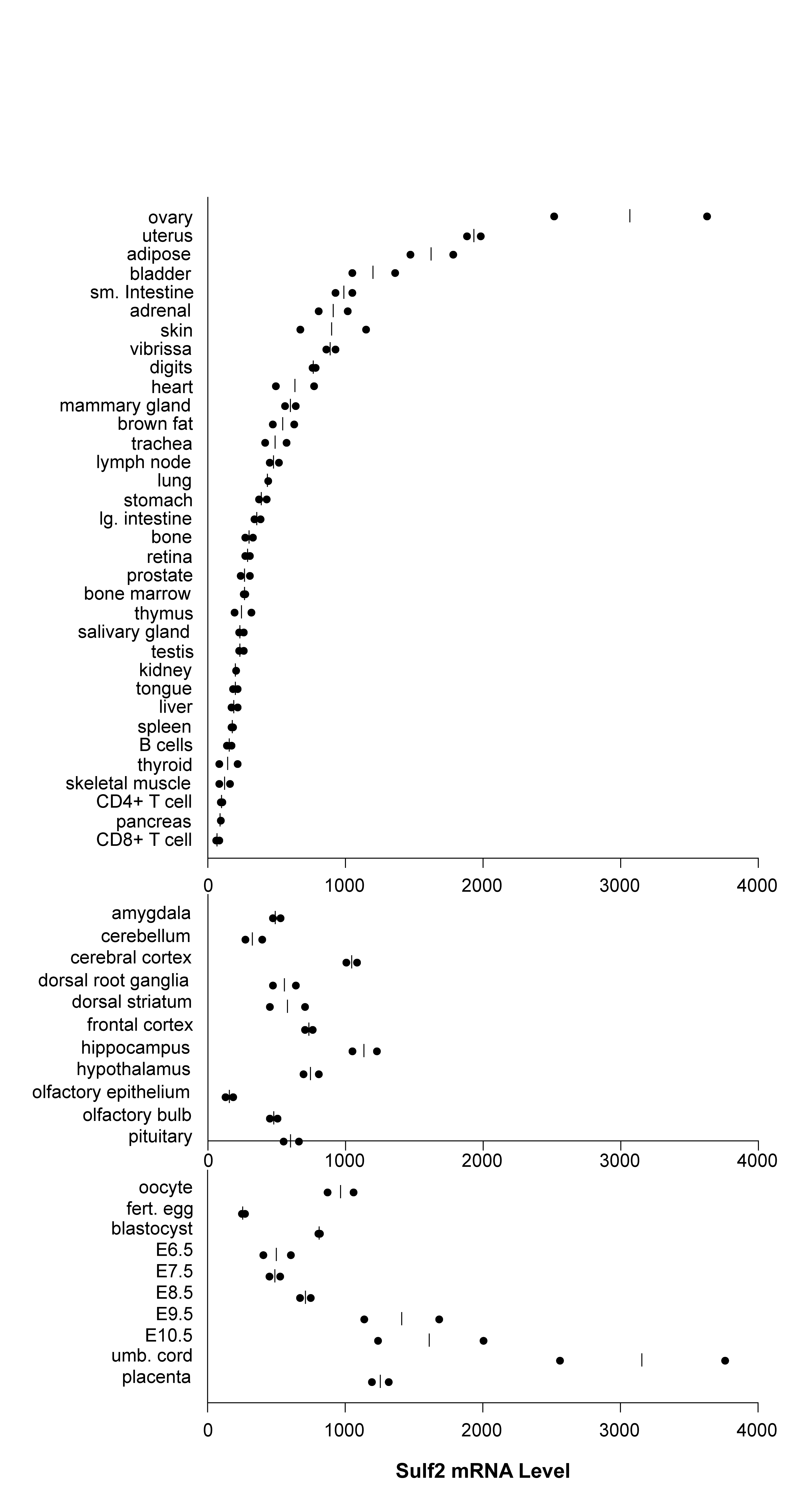

Supplement: Figure S5 — Sulf2 is also expressed in numerous tissues in adult and developing mice. Expression of Sulf2 in tissues derived from duplicate wild-type C57BL/6 adults (upper two panels) and embryonic samples (lower panel), as determined by consulting the microarray analysis submitted to Gene Expression Omnibus (GEO) with accession number GSE1133. Expression values are shown for probeset gnflm29631_a_at on the custom Affymetrix chip [25]. Each point refers to a different sample; the lines represent the mean expression value for each tissue. (0.61 MB TIF) [file pone.0000575.s005.tif]

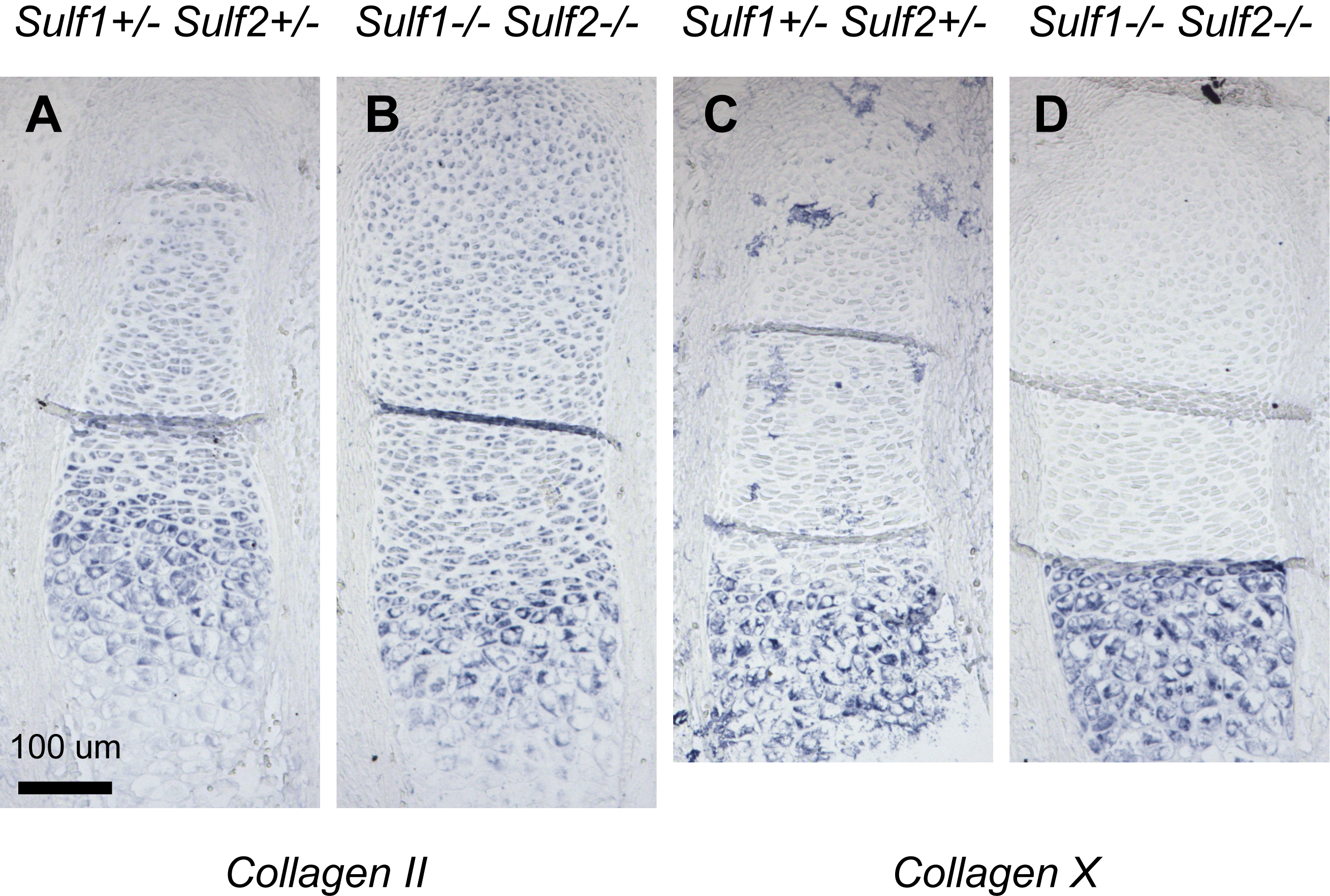

Supplement: Figure S6 — In situ hybridization of bone differentiation markers in sulfatase-deficient embryos. Collagen II (A, B) and Collagen X (C, D) expression were assessed in sections of distal ulnas of control Sulf1 +/− Sulf2 +/− (A, C) and Sulf1 −/− Sulf2 −/− (B, D) E15.5 embryos. (10.15 MB TIF) [file pone.0000575.s006.tif]
